# Age‐related dysregulation of the retinal transcriptome in African turquoise killifish

**DOI:** 10.1111/acel.14192

**Published:** 2024-05-14

**Authors:** Steven Bergmans, Nicole C. L. Noel, Luca Masin, Ellen G. Harding, Aleksandra M. Krzywańska, Julie D. De Schutter, Rajagopal Ayana, Chi‐Kuo Hu, Lut Arckens, Philip A. Ruzycki, Ryan B. MacDonald, Brian S. Clark, Lieve Moons

**Affiliations:** ^1^ Department of Biology, Animal Physiology and Neurobiology Division, Neural Circuit Development & Regeneration Research Group KU Leuven, Leuven Brain Institute Leuven Belgium; ^2^ University College London, Institute of Ophthalmology London UK; ^3^ John F Hardesty, MD Department of Ophthalmology and Visual Sciences Washington University School of Medicine Saint Louis Missouri USA; ^4^ Department of Biology, Animal Physiology and Neurobiology Section, Laboratory of Neuroplasticity and Neuroproteomics KU Leuven, Leuven Brain Institute Leuven Belgium; ^5^ Department of Biochemistry and Cell Biology Stony Brook University Stony Brook USA; ^6^ Department of Genetics Washington University School of Medicine Saint Louis Missouri USA; ^7^ Department of Developmental Biology Washington University School of Medicine Saint Louis Missouri USA; ^8^ Center of Regenerative Medicine Center of Regenerative Medicine, Washington University School of Medicine Saint Louis Missouri USA

**Keywords:** ageing, gliosis, inflammageing, neurodegeneration, *Nothobranchius furzeri*, oxidative stress, retina, transcriptomics

## Abstract

Age‐related vision loss caused by retinal neurodegenerative pathologies is becoming more prevalent in our ageing society. To understand the physiological and molecular impact of ageing on retinal homeostasis, we used the short‐lived African turquoise killifish, a model known to naturally develop central nervous system (CNS) ageing hallmarks and vision loss. Bulk and single‐cell RNA‐sequencing (scRNAseq) of three age groups (6‐, 12‐, and 18‐week‐old) identified transcriptional ageing fingerprints in the killifish retina, unveiling pathways also identified in the aged brain, including oxidative stress, gliosis, and inflammageing. These findings were comparable to observations in the ageing mouse retina. Additionally, transcriptional changes in genes related to retinal diseases, such as glaucoma and age‐related macular degeneration, were observed. The cellular heterogeneity in the killifish retina was characterized, confirming the presence of all typical vertebrate retinal cell types. Data integration from age‐matched samples between the bulk and scRNAseq experiments revealed a loss of cellular specificity in gene expression upon ageing, suggesting potential disruption in transcriptional homeostasis. Differential expression analysis within the identified cell types highlighted the role of glial/immune cells as important stress regulators during ageing. Our work emphasizes the value of the fast‐ageing killifish in elucidating molecular signatures in age‐associated retinal disease and vision decline. This study contributes to the understanding of how age‐related changes in molecular pathways may impact CNS health, providing insights that may inform future therapeutic strategies for age‐related pathologies.

## Graphical abstract

Transcriptomic analysis of the ageing killifish retina showed a marked upregulation of processes observed to be altered in the aged human retina and brain, including oxidative stress, gliosis, and inflammageing. Single‐cell RNA sequencing unveiled the cellular heterogeneity of the killifish retina, while the integration of both bulk and single‐cell RNAseq data highlighted transcriptomic dysregulation in the aged retina.

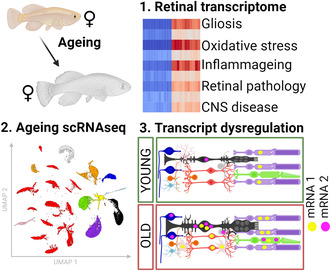

**Abbreviations**
AMD: age‐related macular degeneration
cL15: complete Leibovitz's 15
CNS: central nervous system
DEPC: diethyl pyrocarbonate
FC: fold change
FDR: false discovery rate
GCL: ganglion cell layer
GO: gene ontology
GS: glutamine synthetase
HCR: hybridisation chain reaction
IHC: immunohistochemistry
ILM: inner limiting membrane
INL: inner nuclear layer
IPL: inner plexiform layer
n: number of biological replicates
NFL: nerve fibre layer
OLM: outer limiting membrane
ONL: outer nuclear layer
OPL: outer plexiform layer
PBS: phosphate buffered saline
PC: principal component
PCA: principle component analysis
PFA: paraformaldehyde
PRL: photoreceptor layer
RBC: red blood cell
RIN: RNA integrity number
RNAseq: RNA sequencing
RPE: retinal pigment epithelium
RT: room temperature
scRNAseq: single‐cell RNA sequencing
UMAP: uniform manifold approximation and projection
Unch: unchanged
w: weeks

## 1 INTRODUCTION

The central nervous system (CNS) is vulnerable to the accumulation of age‐related pathologies that lead to progressive, irreversible disease. Ageing is a major risk factor for many retinal neurodegenerative diseases that severely impact visual function and quality of life (Congdon, 2004; Enoch et al., 2019; Swenor et al., 2020). Retinal degenerative diseases, including glaucoma and age‐related macular degeneration (AMD), are becoming more prevalent as life expectancy increases (Klein & Klein, 2013). There are currently no treatments available to restore vision after retinal degeneration, nor targeted therapies to prevent degeneration and related vision loss. Therefore, it is essential to determine the physiological and molecular consequences of ageing on retinal homeostasis and identify mechanisms underpinning age‐related retinal dysfunction.

Small animals such as mice, rats, and zebrafish have been extensively utilised to study fundamental mechanisms underlying different retinal neurodegenerative diseases (Collin et al., 2020; Koh et al., 2019; Niwa et al., 2016; Noel et al., 2022; Veys et al., 2021). Zebrafish naturally undergo age‐related retinal changes that include photoreceptor degeneration, gliosis, and vision loss (Martins et al., 2022; Van houcke et al., 2019). Unfortunately, ageing studies are challenging in these model organisms due to their relatively long lifespan (>2 years) (Kim et al., 2016). Invertebrate gerontology models such as *Caenorhabditis elegans* or *Drosophila melanogaster* have short lifespans of a few weeks but do not allow for comparable study of the retina as they lack a similar system (structure and cell types). This demonstrates the pressing need for a genetically tractable vertebrate model with a condensed lifespan to facilitate higher throughput research into retinal ageing.

The short‐lived African turquoise killifish (*Nothobranchius furzeri*) is emerging as a leading vertebrate genetic model system for ageing studies (Kim et al., 2016; Moses et al., 2023). The GRZ strain has the shortest lifespan in captivity amongst vertebrates, with a 4–6 month life expectancy (Kim et al., 2016). Killifish show macroscopic ageing traits like those observed in humans, such as depigmentation, spinal curvature, muscle atrophy, and cognitive decline (Cellerino et al., 2016; Kim et al., 2016), as well as molecular hallmarks including oxidative stress, cellular senescence, altered cellular communication, and stem cell exhaustion (Baumgart et al., 2014; Valenzano et al., 2006).

Our recent characterisations of the killifish visual system identified several ageing hallmarks in the retina and optic tectum, including increased oxidative stress, DNA damage, inflammageing, stem cell depletion, and cellular senescence (Vanhunsel et al., 2021). Interestingly, like the killifish brain (Bagnoli et al., 2022; Matsui et al., 2019), the killifish retina presented signs of thinning and neurodegeneration upon ageing, which were associated with vision loss (Vanhunsel et al., 2021).

We performed unbiased transcriptomics profiling to better understand the molecular signatures of killifish retinal ageing. We first assayed retinal transcriptome dynamics across ageing using bulk RNA‐sequencing (RNAseq), identifying transcriptional shifts from young adult (6‐week‐old) to old (18‐week‐old). We investigated the cell type specificity of ageing phenotypes using single‐cell RNA‐sequencing (scRNAseq), defining retinal cell populations and identifying age‐induced transcriptome changes within individual retinal cell types. We determined that there is age‐associated transcriptome dysregulation across all retinal cell populations, highlighting systemic changes in transcriptional homeostasis that likely contribute to reduced killifish retinal function with age. From these studies, we highlight the utility of killifish for understanding the molecular aetiology of retinal ageing.

## 2 RESULTS

### 2.1 Bulk RNAseq analysis identifies age‐related transcriptional shifts in the killifish retina

**FIGURE 1:**
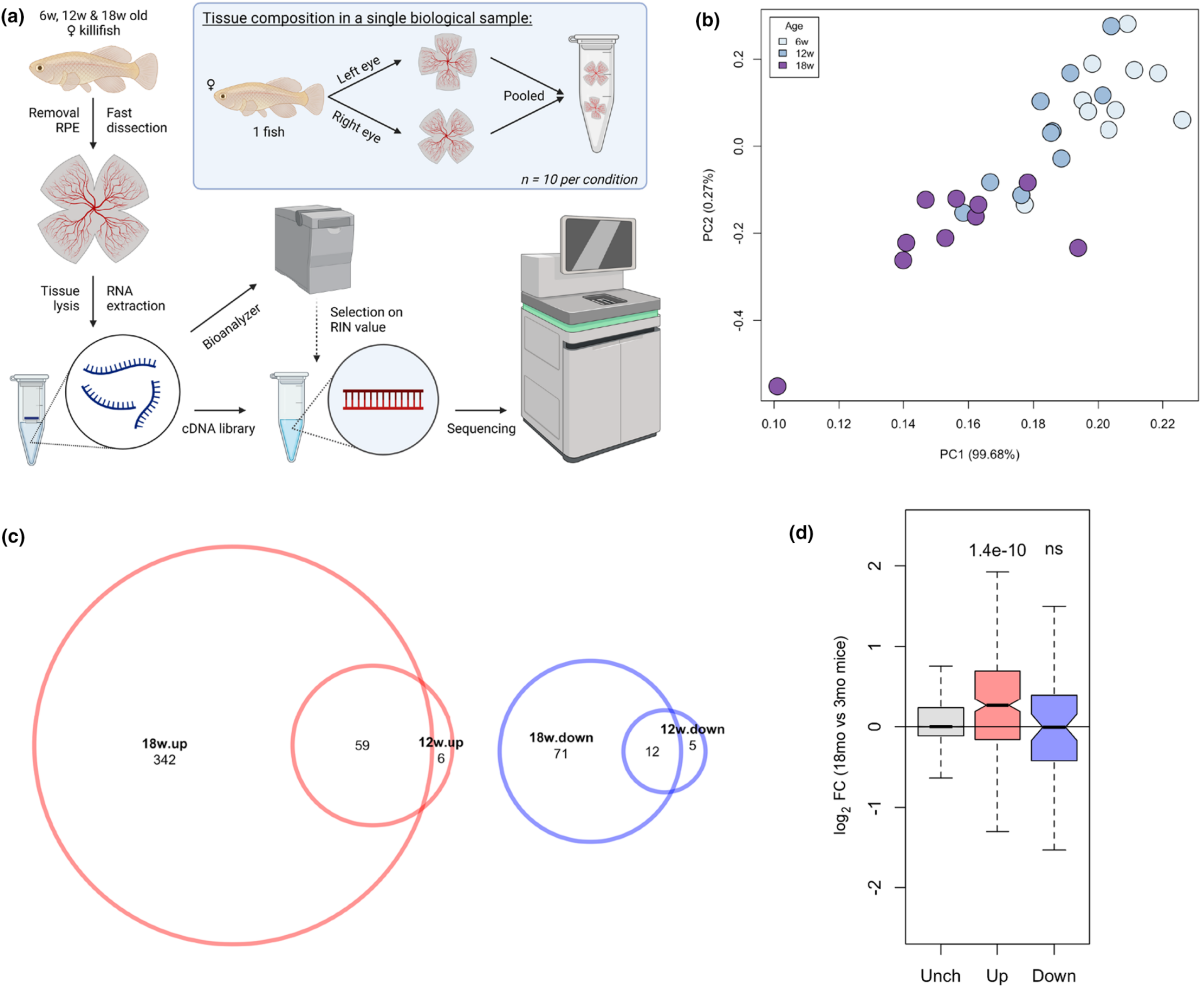
Killifish retinas have age‐related changes in gene expression. (a) Bulk RNAseq experimental setup. Created with BioRender.com. (b) Principal component analysis of 6 (young), 12 (middle‐aged) and 18 weeks of age (old) individual RNAseq samples showing age‐related variance in transcriptome across samples (*n* = 10). (c) Venn diagrams depicting up‐ (red) and down‐regulated (blue) genes between young, middle‐aged, and aged retinas. More upregulated genes than downregulated were identified with age. Differentially expressed genes were identified using following thresholding criteria: FDR ≤0.05 and |log_2_FC| ≥ 1. (d) Boxplots representing the age‐dependent fold change of transcript expression of mouse orthologs (Xu et al., 2022) to up, down, and unchanged sets of differentially expressed killifish genes. The killifish unchanged (grey) and upregulated (red) genes show a similar expression trend as in the ageing mouse retina. This is not observed for the downregulated genes (blue) (Wilcoxon rank‐sum test). FC, fold change; mo, months; ns, not significant; PC, principal component; RIN, RNA integrity number; RPE, retinal pigment epithelium; Unch, unchanged; w, weeks.

We first profiled retinal transcript expression using bulk RNAseq to characterize transcriptional alterations that may underly the killifish ageing phenotypes. We investigated young adult (6‐week‐old), middle aged (12‐week‐old), and old (18‐week‐old) female fish, with 10 biological replicates per age group (Figure 1a). Neural retinas were dissected after retinal pigmented epithelium (RPE) removal and processed for bulk RNAseq. Principal component analysis revealed a gradual shift in transcriptional state that correlated with age (Figure 1b). We identified 65 and 401 upregulated genes between 6‐ and 12‐week‐old, and between 6‐ and 18‐week‐old retinas, respectively, by direct comparison of gene expression across individual ages (cut‐offs: FDR <0.05, log_2_FC ≥ 1). Of these differentially expressed transcripts, 59 were shared across both comparisons (Figure 1c). A smaller number of transcripts showed decreased expression across retinal ageing, with 17 genes having decreased transcript expression between 6‐ and 12‐week‐old and 83 genes between 6‐ and 18‐week‐old retinas, of which 12 genes were shared across age comparisons (log_2_FC <1 and FDR <0.05; Figure 1c). A heatmap and table including all differentially expressed transcripts across retinal ageing are provided in Figure S1 and Table S1. We focused on genes changing in expression between 6‐ and 18‐week‐old samples for subsequent analyses, as these data highlighted the greatest age‐associated transcriptional disparity.

We next compared our dataset to published mouse ageing retinal transcriptomes (3 vs. 18 months old (Xu et al., 2022)) to investigate the similarities between killifish and mammalian retinal ageing. Genes with increased expression during killifish retinal ageing showed a similar trend to their mouse orthologs (Figure 1d). In contrast, genes that decreased in the ageing killifish dataset did not show consistency with the mouse findings. As many genes identified as downregulated in the killifish retina were RPE‐related genes, such as *rpe65a*, *tyr*, and *pmelb* (Table S1), some downregulated genes may be the technical artifact of RPE removal. We observed that young killifish have higher RPE adherence to the neural retina than old killifish, making it more challenging to physically remove the RPE, potentially suggesting age‐related differences in the interaction between photoreceptors and RPE. As a result, relative RPE cell numbers and their transcriptional signatures may be higher in datasets from young animals, resulting in an artifactually perceived downregulation of RPE‐related genes with ageing. Therefore, definitive conclusions concerning RPE‐related genes cannot be drawn here. Gene ontology (GO) term analysis of our upregulated gene dataset revealed pathway enrichments related to oxidative stress (GO:0051775, GO:0034599, and GO:0006979), metabolism (GO:0042572 and GO:0031325), and inflammation (GO:0090025 and GO:0043368); these findings coincide with those of the aged murine retinal transcriptome for pathways correlated to oxidative stress and inflammatory responses (Xu et al., 2022), which had age‐related changes in similar categories. See Table S2 for a list of the GO categories for all up‐ and down‐regulated biological processes and molecular functions.

**FIGURE 2:**
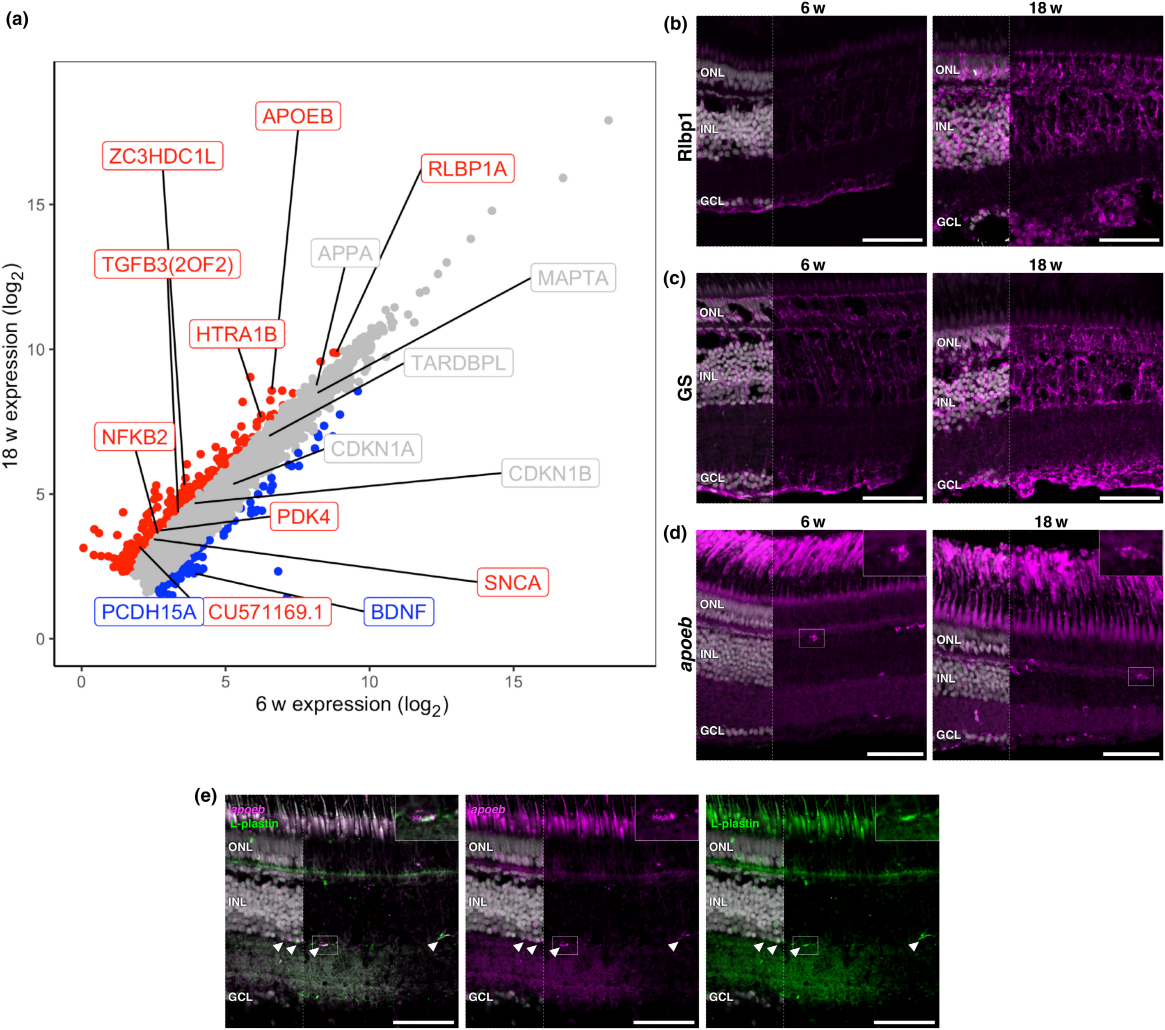
Aged killifish retinas show signs of gliosis, inflammageing, and neurodegeneration detected by bulk RNAseq. (a) Scatter plot showing mean expression of all genes detected by bulk RNAseq across 6 w and 18 w samples. The different colours denote genes that passed FDR and FC thresholds (FDR <0.05 and |log_2_FC| ≥ 1). Genes that are not significantly changed are shown in grey; upregulated genes are in red; downregulated genes in blue. ZC3HDC1L = *optn*, CU571169.1 = *slc7a11*. (b) Staining for Rlbp1 shows a visual increase in Rlbp1 protein expression in aged retinas, corresponding to increased transcript expression in RNAseq. (c) Immunostaining of glutamine synthetase shows expansion of Müller glia cell morphology in 18‐week‐old retinas compared to 6‐week‐old retinas. (d) in situ HCR for *apoeb* in 6‐ and 18‐week‐old retinas highlight an increase in immune cells with age as well as changes in their morphology (insets), signs of inflammageing. (e) Microglia/macrophage‐specific labelling by *apoeb* is confirmed by antibody co‐labelling with the pan‐leukocyte marker L‐plastin (arrowheads). Merged images with nuclei shown in left third of image; remaining image is without nuclei. Scale bars = 50 μm. FC, fold change; FDR, false discovery rate; GCL, ganglion cell layer; GS, glutamine synthetase; HCR, hybridisation chain reaction; INL, inner nuclear layer; ONL, outer nuclear layer; unch, unchanged; w, weeks.

We sought to first validate important processes associated with CNS ageing as well as killifish retinal ageing hallmarks that were previously identified using histological analyses (Mattson & Arumugam, 2018; Vanhunsel et al., 2021). We looked at genes involved in oxidative stress, gliosis, inflammageing, and cellular senescence. Oxidative stress occurs due to the accumulation of reactive oxygen species and is a prominent driver of progressive loss of tissue function during ageing (Liguori et al., 2018). We observed increased transcript levels for the oxidative stress response genes *pdk4* (log_2_FC = 1.16, *p*‐value = 4.98E‐12, FDR = 1.81E‐10) and *slc7a11* (log_2_FC = 1.48, *p*‐value = 6.25E‐15, FDR = 3.62E‐13) in the aged killifish retina (Figure 2a) (Gao et al., 2022; Yan et al., 2023). Furthermore, we detected increased transcript levels for the genes *tgfb3* (log_2_FC = 1.04, *p*‐value = 1.54E‐11, FDR = 5.10E‐10) and *rlbp1a* (log_2_FC = 1.03, *p*‐value = 3.31E‐15, FDR = 2.05E‐13), known to be related to gliosis (Conedera et al., 2021) and Müller glia homeostasis (Vázquez‐Chona et al., 2009), respectively (Figure 2a). Glia become reactive in response to stress/injury, a process referred to as gliosis that is defined by morphological and gene expression changes (Bringmann et al., 2009; Seitz et al., 2013). Using immunohistochemistry (IHC), we correlated the increased *rlbp1a* transcript expression with an appreciable rise in Rlbp1 staining in aged retinas, where signal intensity visually increased within cells exhibiting Müller glia morphology. Furthermore, we observed expansion and elaboration of Müller glia morphology based on Rlbp1 labelling (Figure 2b, Figure S2). This glia hypertrophy was confirmed by immunolabelling for the Müller glia marker glutamine synthetase (GS) (Figure 2c). This persistent activation phenotype in Müller glia might alter their physiological homeostatic function resulting in impaired support of retinal neurons (Bringmann et al., 2006; Nag, 2023; Reichenbach & Bringmann, 2013; Telegina et al., 2018). Additionally, activated Müller glia can alter their expression pattern favouring a pro‐inflammatory secretory phenotype (Bringmann et al., 2009; Clarke et al., 2018; Eastlake et al., 2016), that may further affect neuronal integrity and functioning. Furthermore, the aged immune system is known to undergo inflammageing, where it alters cell number, morphology, and secretory profile (Godbout & Johnson, 2009; Vanhunsel et al., 2021). Our bulk RNAseq data revealed an upregulation of inflammageing markers such as *nfkb2* (log_2_FC = 1.07, *p*‐value = 4.78E‐10, FDR = 1.174E‐8) and *apoeb* (log_2_FC = 1.99, *p*‐value = 8.01E‐49, FDR = 6.09E‐46) in old retinas (Figure 2a) (García‐García et al., 2021; Godbout & Johnson, 2009). Assessments using in situ hybridization chain reaction (HCR) for *apoeb* highlighted a visible increase in the amount of *apoeb*‐positive cells in the aged retina as well as differences in morphology between 6 and 18 weeks (Figure 2d). We confirmed that *apoeb* labelled immune cells using IHC for the pan‐leukocyte marker L‐plastin (Figure 2e). Thus, we observed increased oxidative stress levels as well as Müller glia and microglia reactivity in the aged killifish retina, consistent with previous reports (Vanhunsel et al., 2021). However, unlike our previous study characterizing senescence markers (*cdkn1a/*p21 and *cdkn1b/*p27) by quantitative reverse‐transcription PCR, we did not observe significant transcriptional changes for these genes between young and old neural retinas in the bulk RNAseq (Figure 2a).

We next investigated whether retinal degeneration‐associated genes for human retinal diseases, such as glaucoma and AMD, were changing across age. While pathogenic *OPTN* variants are known to result in hereditary forms of glaucoma (Rezaie et al., 2002), increased *OPTN* levels are associated with neuroprotective processes (Markovinovic et al., 2018; Weil et al., 2018). Our dataset revealed an age‐related increase in *optn* transcript levels (log_2_FC = 1.79, *p*‐value = 4.36E‐33, FDR = 1.43E‐30; Figure 2a), but the effect of this increase remains elusive. Brain‐derived neurotrophic factor (BDNF) is important for neuronal homeostasis and survival (Azman & Zakaria, 2022; Lima Giacobbo et al., 2019). We observed a decrease in *bdnf* (log_2_FC = −1.87, *p*‐value = 1.20E‐26, FDR = 2.66E‐24) expression with age (Figure 2a), which may result in a heightened risk of developing retinal pathology. Indeed, BDNF is of interest for treating neurodegenerative diseases such as glaucoma and is reduced in glaucomatous eyes (Gupta et al., 2014; Osborne et al., 2018). Variants in the *HTRA1* promoter and gene increase the risk for developing AMD (DeAngelis et al., 2008; Friedrich et al., 2015; Tam et al., 2008; Yang et al., 2006). Interestingly, Htra1 protein levels increased with age in the zebrafish retina and elevated *htra1* transcript levels were observed in a zebrafish photoreceptor degeneration model (Oura et al., 2018). The expression of *htra1b* increased (log_2_FC = 1.40, *p*‐value = 1.86E‐25, FDR = 3.71E‐23) in the aged killifish retina (Figure 2a), which may be indicative of photoreceptor dysfunction. Pathogenic variants in *PCDH15* cause Usher syndrome, a condition characterized by hearing and vision loss (Ahmed et al., 2001). *pcdh15a* expression decreased (log_2_FC = −1.11, *p*‐value = 7.06E‐8, FDR = 9.25E‐7) with age in the killifish retina (Figure 2a), which may also suggest degradation of photoreceptor health. Thus, genes known to be associated with retinal degeneration show age‐dependent expression changes in the killifish retina, highlighting the value of the killifish retina as an ageing model.

We next examined changes in expression of genes associated with degenerative disease, as pathological hallmarks of neurodegenerative disorders can also manifest within the retina (London et al., 2013; Veys et al., 2019). Interestingly, we observed increased transcript levels of the gene encoding alpha‐synuclein (*snca*), known to be a key player in Parkinson's disease and upregulated in the killifish brain (Matsui et al., 2019), in the aged killifish retina (log_2_FC = 1.13, *p*‐value = 1.28E‐10, FDR = 3.51E‐9; Figure 2a). In contrast, we did not observe differential expression of Alzheimer‐related genes *mapta* (encoding tau) or *appa* (encoding amyloid beta precursor protein), nor the frontotemporal dementia/amyotrophic lateral sclerosis gene *tardbp* (encodes Tdp‐43). This could indicate that features reported in the aged killifish brain, such as amyloid beta‐plaques and Tdp‐43 stress granules (Bagnoli et al., 2022; Bergmans, Raes, et al., 2023; de Bakker & Valenzano, 2023; Louka et al., 2022; Matsui et al., 2019), are not manifesting in the ageing killifish retina, although investigation at the protein level would be required to determine this.

In conclusion, bulk RNAseq analysis of the aged killifish retina identified conserved age‐related changes in genetic pathways associated with retinal ageing and neurodegenerative disease.

### 2.2 scRNAseq catalogues all cell types in the killifish retina

**FIGURE 3:**
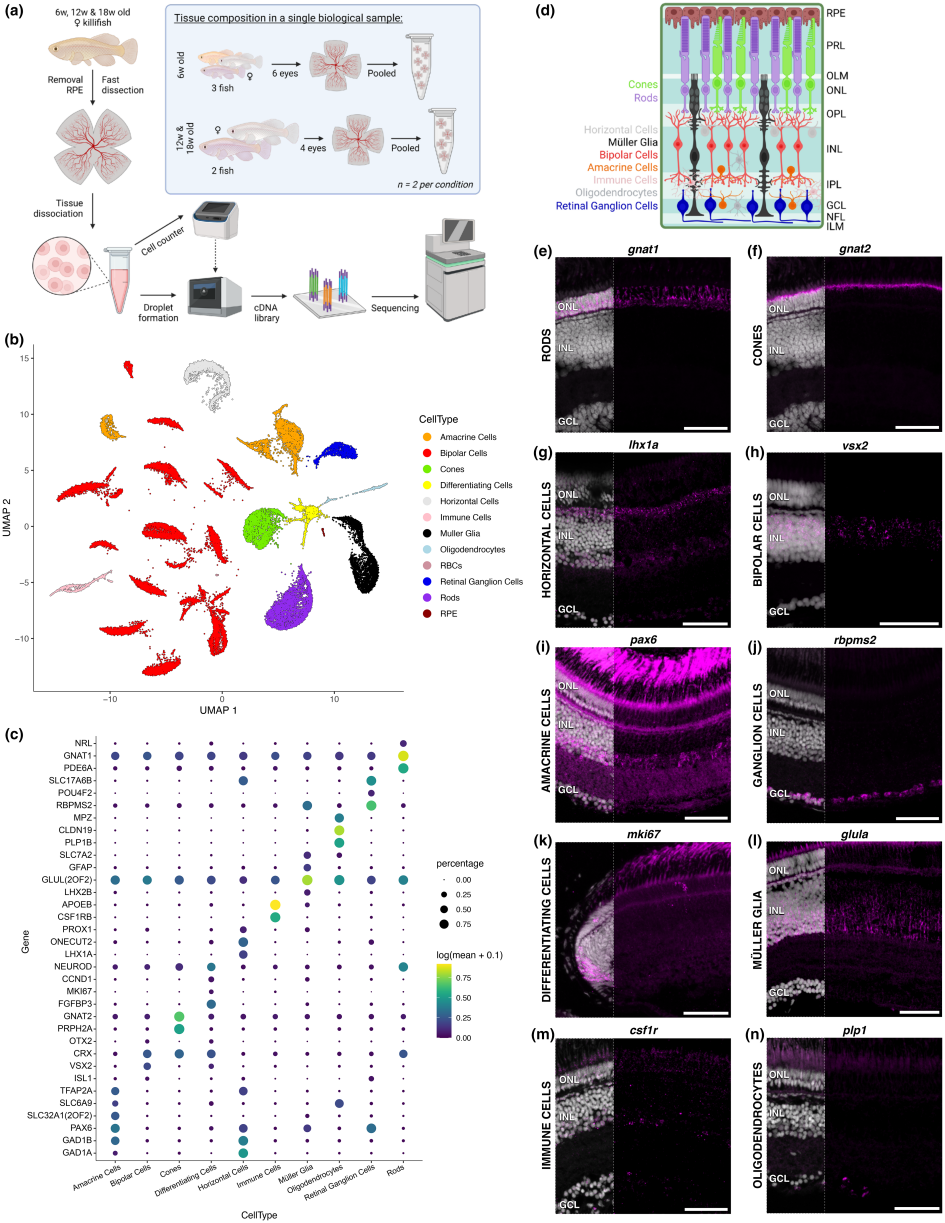
scRNAseq identifies neuronal and glial cell types within the killifish retina. (a) scRNAseq experimental setup. Created with BioRender.com. (b) UMAP dimension reduction of the retina scRNAseq dataset with clusters coloured by annotated retinal cell type. (c) Dot plot showing the specificity of marker genes within individual retinal cell types. The size of the dot represents the percentage of cells within the population expressing transcripts for the gene, while the colour indicates the average expression across individual cells. (d) Schematic representation of the cell types identified in the killifish retina and where they reside within the retinal architecture. Cell types are coloured‐coded to the UMAP in (b). Created with BioRender.com. (e–n) Spatial validation of cell type marker genes via in situ HCR confirms the identification for every retinal cell type. Merged images with nuclei shown in left third of image. Scale bars = 50 μm. GCL, ganglion cell layer; HCR, hybridisation chain reaction; ILM, inner limiting membrane; INL, inner nuclear layer; IPL, inner plexiform layer; NFL, nerve fibre layer; OLM, outer limiting membrane; ONL, outer nuclear layer; OPL, outer plexiform layer; PRL, photoreceptor layer; RBC, red blood cell; RPE, retinal pigment epithelium; UMAP, uniform manifold approximation and projection; w, weeks.

Age‐associated retinal diseases, including AMD and glaucoma, selectively impact individual cell types leading to their progressive loss. This is likely the result of a complex interplay between genetics and the environment, as disease risk‐associated variants are not wholly restricted to genes expressed only in cell type(s) primarily affected by disease pathology and can underlie different diseases in a variant‐specific manner. For instance, *OPTN* is not only expressed in retinal ganglion cells, but is also found in a subset of retinal interneurons, the brain, and non‐neural tissues such as the heart (Kroeber et al., 2006; Rezaie & Sarfarazi, 2005). However, glaucoma‐associated disease variants lead to retinal ganglion cell death (Rezaie et al., 2002). One hypothesis is that the ageing process and related stresses causes asymmetric alterations in gene regulation across cell types. Alternatively, ageing may manifest as a more general process, resulting in global changes in gene expression across all cell types, with environmental factors contributing to disease onset and/or severity. To investigate how gene expression changes across retinal ageing affect transcript expression within individual cell types, we performed scRNAseq age‐matched to the bulk RNAseq experiments (Figure 3a). 10× Genomics 3′ sequencing was performed on the three age groups with two biological replicates per age (6‐, 12‐, and 18‐week‐old animals), capturing 37,243 cells across the ages with an average transcript count of 3311 ± 2672 per cell (Figure S3).

Dimension reduction resulted in discrete clustering of cell populations (Figure 3b, Figure S3b). Each cluster was comprised of cells from each biological replicate and timepoint except for clusters 23 and 29 (28 and 27 cells, respectively; Figure S3c). Known marker genes for major cell classes were used to distinguish retinal cell types, including cone photoreceptors (*gnat2*), rod photoreceptors (*nrl* and *rho*), horizontal cells (*lhx1a*), bipolar cells (*vsx2*), Müller glia (*lhx2b* and *aqp1a.1*), amacrine cells (*tfap2b* and *pax6*), and retinal ganglion cells (*pou4f2* and *gap43*) (Figure 3b,c, Figure S4). We also observed clusters containing immune cells (microglia/macrophages; *aif1*, *c1qc*), red blood cells (*Nfu_p_1_007730*, encoding haemoglobin subunit alpha A), and RPE cells (*rpe65*) (Figure S5), as well as a cluster containing cells marked by proliferative markers (*ccnd1*) and markers of retinal neurogenesis (*neurod*; differentiating cells) (Figure 3b, Figure S4). All cell type identities were maintained across ageing, and while there may be shifts in cell proportion between the samples investigated, we did not observe substantial age‐dependent loss of individual cell types (Figure S3d). Previous morphological studies on killifish retina found that there was an age‐related increase in immune cells and a decrease in stem cells/differentiating cells (Vanhunsel et al., 2021). The cell proportions estimated from the scRNAseq results coincide with these findings (Figure S3e)—however, capture efficiency biases of individual cell types cannot be ruled out. Notably, GO term analysis of the bulk RNAseq dataset also unveiled negative regulation of cell population proliferation (GO:0008285) (Table S2), which together with a decline in stem cells might suggest adverse implications for neuronal regeneration in the aged killifish retina.

Cell type cluster markers (neurons, glia, and immune cells) were validated on tissue using in situ HCR (Figure 3e–n), (Figure S6a–i), confirming the basic vertebrate retinal architecture (Figure 3d). Briefly, the retina is comprised of three nuclear layers: the outer nuclear layer (ONL), inner nuclear layer (INL), and ganglion cell layer (GCL), separated by synaptic layers, the outer and inner plexiform layers (OPL and INL) (Yamagata et al., 2021). General photoreceptor markers (*crx*) (Figure S6a) and photoreceptor subtype‐specific labelling for rods (*gnat1* and *nrl*) (Figure 3e, Figure S6b) and cones (*gnat2*) (Figure 3f) were confined to the outer retina, with rod nuclei sitting below cone nuclei. Horizontal (*lhx1a* and *tfap2a*) (Figure 3g, Figure S6c), bipolar (*vsx2* and *otx2*) (Figure 3h, Figure S6d), and amacrine (*pax6* and *slc32a1b*) (Figure 3i, Figure S6e) cells were observed in the INL and localized to their specific sublayers: the apical INL, dispersed throughout the INL, and along the basal INL, respectively. Retinal ganglion cells in the GCL were labelled with markers *rbpms2* and *slc17a6b* (Figure 3j, Figure S6f). Differentiating cells, marked by *stmn1a, fgfb3*, and *mki67* (Figure 3k, Figure S6g), were present in the ciliary marginal zone, the neurogenic niche at the retinal periphery. Müller glia (*glula* and *rlbp1a*) showed characteristic radial fibre morphology with their nuclei confined to the INL (Figure 3l, Figure S6h), while *gfap* signal was restricted to their end feet (Figure S6h). Immune cells, marked by *csf1r*, lined the IPL (Figure 3m), as expected for resting microglia (Murenu et al., 2022). Notably, in contrast to mammals, we identified oligodendrocytes (*plp1b* and *cldn19*), highly abundant in the optic nerve head (Figure S6i) and sparsely distributed throughout the retina in the GCL (Figure 3n, Figure S6i) and the INL (Figure S6i) (Nakazawa et al., 1993; Santos‐Ledo et al., 2023).

**FIGURE 4:**
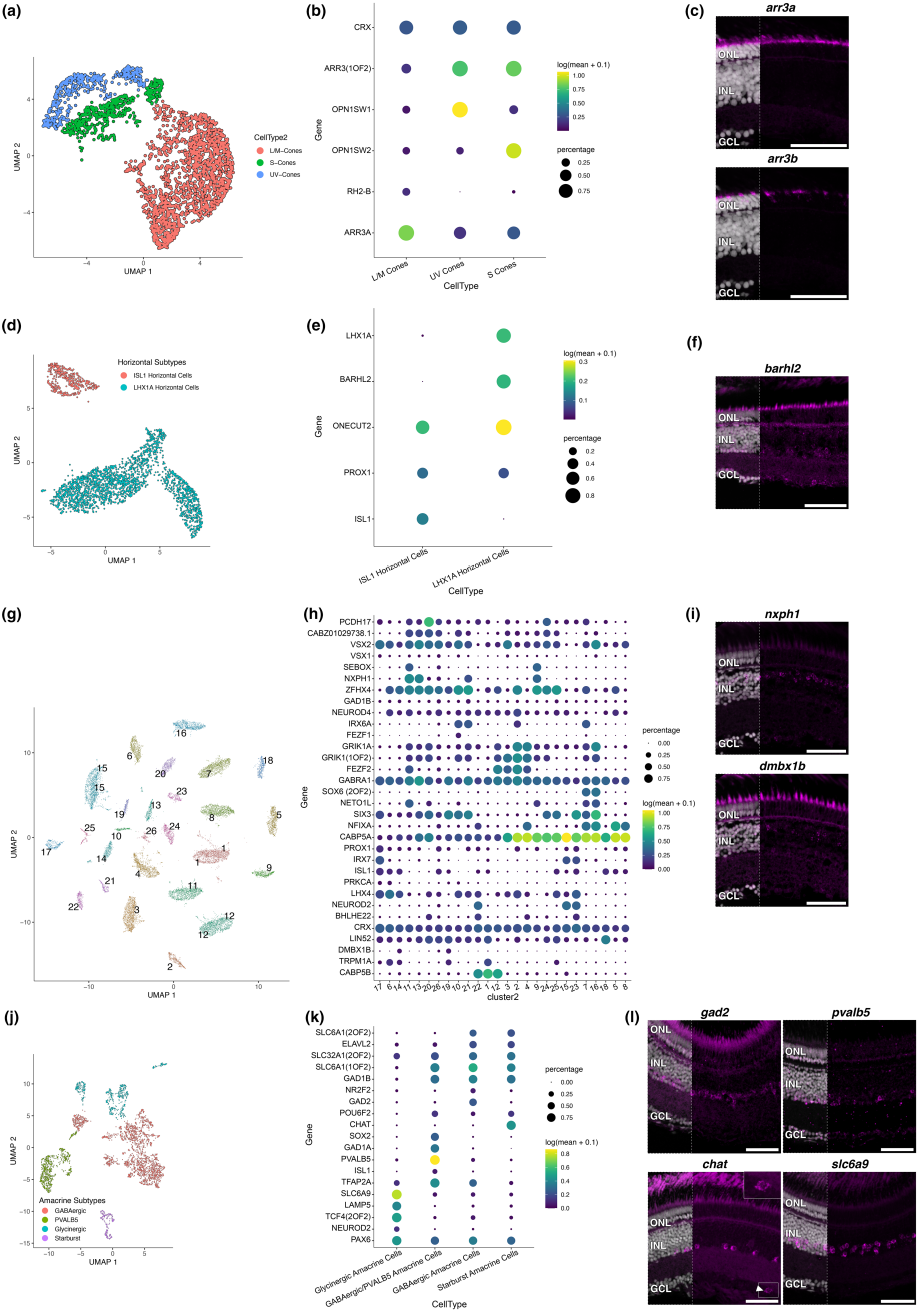
Validation of cell subtypes for specific killifish retinal populations. UMAP dimension reduction shows the subclustering of the photoreceptors (a), horizontal cells (d), bipolar cells (g), and amacrine cells (j). Cell type‐specific markers are shown as dot plots (b, e, h, k). Dot size shows the percentage of cells expressing the marker gene while the colour indicates the mean transcript expression. in situ HCRs of subtype markers for photoreceptors (c), horizontal cells (f), bipolar cells (i), and amacrine cells (l) distinguish specific retinal cell subtypes. A displaced starburst amacrine (*chat+*) is highlighted with an inset box and arrowhead (l). Merged images with nuclei shown in left third of image. Scale bars = 50 μm. GCL, ganglion cell layer; HCR, hybridisation chain reaction; INL, inner nuclear layer; ONL, outer nuclear layer; UMAP, uniform manifold approximation and projection.

For retinal cell types showing heterogeneous clustering in the UMAP, we identified and validated markers that distinguished subtypes when subclustering resolution permitted. We further investigated the expression of subcluster marker genes in the other identified cell types (Figure S7). We subclustered cone photoreceptors into L/M, S, and UV cones (Figure 4a,b). L and M cones are often physically fused as double cones in fishes (Siebeck et al., 2014), preventing effective dissociation and likely resulting in the mixed L/M cluster. S and UV cones labelled for *arr3b* and L/M cones labelled with *arr3b* via in situ HCR, showing similar *arr3* orthologue expression patterns as in zebrafish (Renninger et al., 2011). Two major horizontal cell classes were delineated, namely *isl1‐*positive *and lhx1a‐*positive horizontal cells (Figure 4d,e) in situ HCR for *barhl2*, a gene highly enriched in the *lhx1a*‐positive horizontal cell subcluster, labelled horizontal cells as well as cells within the INL amacrine sublayer and the GCL (Figure 4f), as expected from our scRNAseq dataset (Figure S7b). Subclustering of the bipolar cell population led to the identification of 26 clusters, highlighting the heterogeneity of this cell type within the killifish (Figure 4g,h). Labelling of cohorts of bipolar subclusters using *nxph1* and *dmbx1b* showed an apical to basal localisation within the INL, respectively (Figure 4i). Finally, subclustering of the amacrine cell population revealed four major amacrine types: glycinergic, GABAergic/parvalbumin, GABAergic, and starburst (Figure 4j,k). These amacrine cell types were spatially validated using in situ HCR for *slc6a9*, *pvalb5*, *gad2*, and *chata*, respectively (Figure 4l). Additional markers for glycinergic amacrines (*lamp5* and *tcf4*) are provided in Figure S6e. Interestingly, a visually appreciable difference in cell density could be observed for the amacrine subtypes, where parvalbumin and starburst amacrine cells seem less prevalent compared to GABAergic and glycinergic amacrines. Of note, we were also able to identify displaced starburst amacrines in the GCL (Figure 4l).

In conclusion, we have generated the first molecular survey of the cellular heterogeneity of the killifish retina, identifying all major cell classes typical of a vertebrate retina.

### 2.3 Integration of bulk and single‐cell RNAseq reveals cellular specificity and age‐related dysregulation of retinal gene expression

We confirmed the robustness of the age‐dependent transcriptomic signatures across RNA profiling techniques through comparisons of gene expression changes from the bulk RNAseq within the scRNAseq data (Figure S8). The upregulated and unchanged gene sets detected by bulk RNAseq showed similar age‐dependent expression changes in the scRNAseq dataset, although this was not observed for the downregulated genes (Figure S8a,c).

**FIGURE 5:**
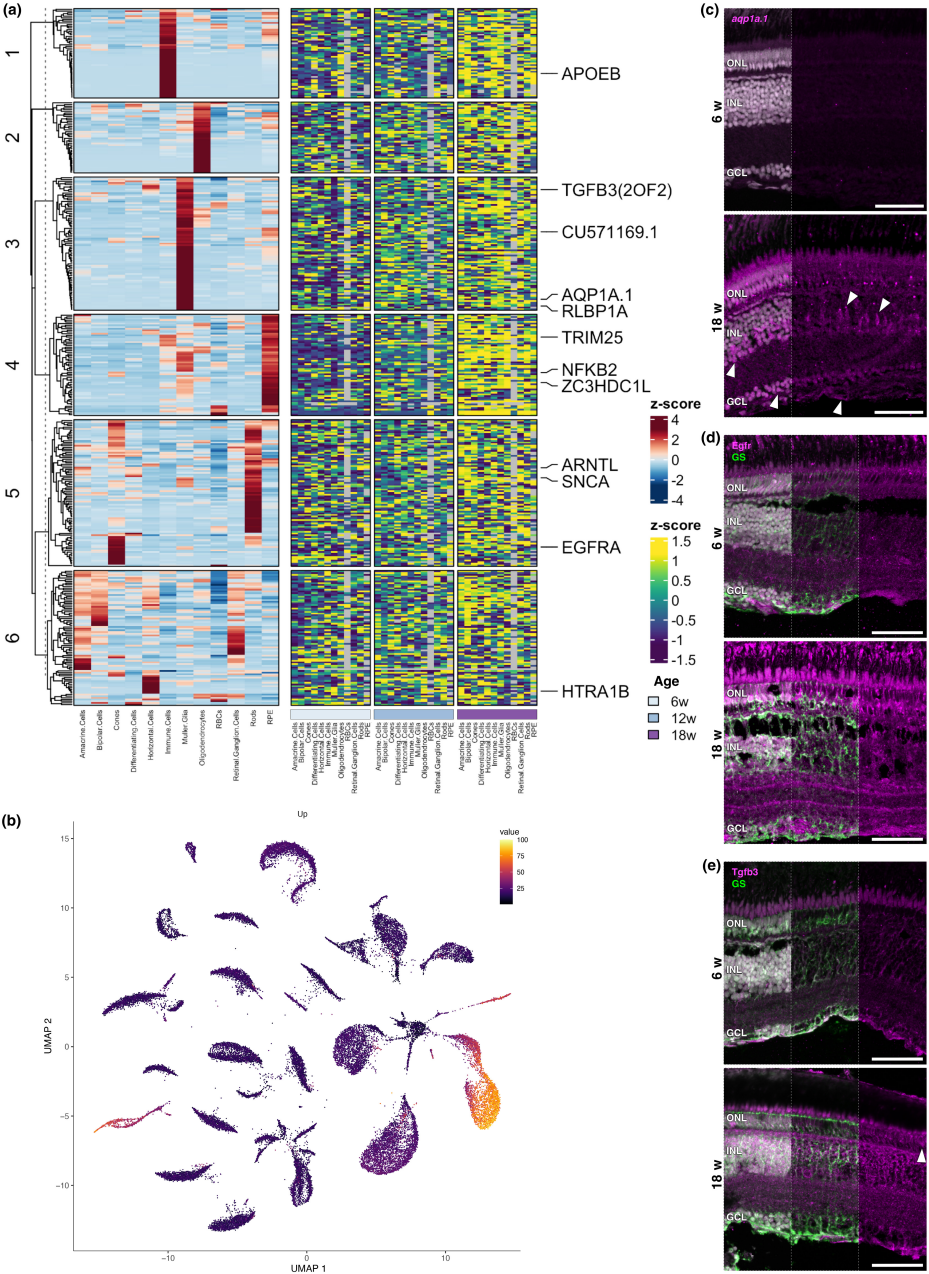
Killifish display age‐associated transcriptional dysregulation. (a) Heatmaps of data integrated from the bulk and scRNAseq. Genes that increase with age are typically expressed in a variety of retinal cell types, with roughly half of the transcripts displaying enriched expression within glial/immune cells (left). With age, the genes increase in expression across numerous cell types (right). Several genes of interest are highlighted. ZC2HDC1L = *optn*, CU571169.1 = *slc7a11*. (b) UMAP showing that the genes that increase with age are overall most highly expressed in Müller glia, immune cells, and oligodendrocytes. (c–e) Spatial validation of upregulated genes; total merge with nuclei shown in left third of image. (c) Transcript for the Müller glia enriched gene *aqp1a.1* is detected in many cell types at 18 weeks by in situ HCR, including ganglion cells and photoreceptors. (d) Immunolabelling for Egfr and GS shows that Egfr is primarily in Müller glia in young retinas. In old retinas there is increased Egfr in the GCL and nerve fibre layer. Middle: Egfr + GS merge without nuclei; right: Egfr only. (e) Antibody labelling for Tgfb3 and GS shows that Tgfb3 protein is restricted to Müller glia in young retinas but appears more globally expressed in old retinas. Middle: Tgfb3 + GS without nuclei; right: Tgfb3 only. Scale bars = 50 μm. GCL, ganglion cell layer; GS, glutamine synthetase; HCR, hybridisation chain reaction; INL, inner nuclear layer; ONL, outer nuclear layer; RBCs, red blood cells; RPE, retinal pigment epithelium; UMAP, uniform manifold approximation and projection; w, weeks.

Next, we sought to examine the cell‐type specificity of the age‐dependent differentially expressed transcripts identified in our bulk RNA‐sequencing experiments through integration of the bulk and scRNAseq dataset (Figure 5a, left; Figure S9a, left). A significant fraction (more than half) of the age‐dependent, downregulated transcripts from the bulk RNAseq analysis displayed enriched expression within RPE cells (Figure S9). For the upregulated genes, nearly half of the genes showed glial/immune enrichment (Figure 5a, left); this is notable as these cell types represent less than a third of the cells in the single‐cell dataset and in the retina (Figure S3e). The aggregate of transcripts that increased expression with ageing were most highly expressed in glia and immune cells (Figure 5b).

As our scRNAseq dataset is age‐matched to bulk RNAseq samples, we plotted the relative expression of each gene within each cell type across time (Figure 5a, right; Figure S8a, right). Strikingly, for genes that showed expression highly enriched within specific cell types when evaluating the dataset as a whole (Figure 5a, left), we observed increased relative expression within many, if not all, retinal cell types of the aged killifish retina (Figure 5a, right). One prominent example was *trim25*. While its general expression is characteristic of immune and RPE cells (Figure 5a, left), in aged individuals all retinal cell types showed increased *trim25* transcript level as compared to their young counterparts (Figure 5a, right). Similarly, we observed that *snca* displayed enriched expression in rod photoreceptors (Figure 5a, left), but during ageing, relative *scna* expression increased in all other retinal cell types compared to their younger counterparts (Figure 5a, right). The Müller glia‐enriched gene *rlbp1a* (Figure 5a, left) displayed major dysregulation in 18‐week‐old retinas, with augmented expression in rods, cones, horizontal cells, Müller glia, retinal ganglion cells, differentiating cells, immune cells, and oligodendrocytes (Figure 5a, right). Of note, while we observed increased *rlbp1a* expression in atypical cell types at the transcript level, we did not observe this at the protein level; Rlbp1 antibody staining was confined to Müller glia in the neural retina and showed glial expansion at 18 weeks (Figure 2b). This discrepancy between Rlbp1 immunolabelling and *rlbp1a* transcript expression may be due to low expression levels in non‐Müller glia resulting in undetectable antibody labelling, or it could be due to a decoupling between translation and transcription processes. Conversely, *arntl* expression did not show a clear confinement to a single retinal cell type (Figure 5a, left), but displayed increased expression in amacrine cells, bipolar cells, cones, immune cells, and oligodendrocytes upon ageing. Notably, this transcriptional dysregulation shown for many of the upregulated transcripts was not prominently observed for downregulated genes (Figure S9a, right).

To validate the observed age‐dependent spatial alterations of genes across cell types, we performed in situ HCR or IHC to examine changes in localisation of selected genes. Aqp1 is associated with the oxidative stress response and the *aqp1a.1* transcript was enriched in Müller glia compared to the other cell types (Figure 5a, left). Upon ageing, transcript levels increased in multiple cell types (Figure 5a, right). In agreement, in situ HCR did not show appreciable *aqp1a.1* signal in 6‐week‐old retinas, presumably due to low expression, but in aged fish *aqp1a.1* expression appeared within Müller glia as well as in amacrine or retinal ganglion cells in the INL and GCL (Figure 5c). scRNAseq data confirmed a relative increase from 6 to 18 weeks of age of *aqp1a.1* expression in amacrine cells (1.24‐fold), Müller glia (1.69‐fold), and retinal ganglion cells (2.11‐fold). However, *aqp1a.1* expression displayed >100‐fold enrichment in Müller glia compared to other retinal cell types across the ages. Egfr is known to promote glial and progenitor cell proliferation (Yoo et al., 2021), but its transcript was overall most highly expressed in cone photoreceptors in our scRNAseq dataset (Figure 5a, left). Upon ageing, *egfr* transcripts showed increased levels in cones, differentiating cells, horizontal cells, Müller glia, and oligodendrocytes (Figure 5a, right). IHC for Egfr revealed induced protein expression across all retinal layers in aged retinas (Figure 5d), confirming our scRNAseq data. This dysregulated expression pattern was, however, not consistent for all genes. *tgfb3*, for instance, which was most enriched within Müller glia (Figure 5a, left), did not show a global increase in transcript expression across the retinal cell types, but rather a specific increase in rod photoreceptors and immune cells (Figure 5a, right). Nevertheless, Tgfb3 protein expression appeared to globally increase in aged killifish retina, including in the photoreceptor layer (Figure 5e). This discrepancy between our transcriptome data and IHC assessment might be due to transcriptional‐translational decoupling, which has been reported in the killifish brain (Kelmer Sacramento et al., 2020).

Altogether, our findings suggest a global age‐dependent phenotype at the transcriptional level. We observe that differentially expressed transcripts within bulk RNA‐seq experiments in aggregate, show consistent, age‐dependent upregulation across cell types (Figure S7). These transcripts retain cell type enrichment between young and aged cell types, but we begin to observe, for example, age‐dependent increases in glial‐enriched genes associated with oxidative stress and/or gliosis within aged neurons. We interpret these changes as an age‐dependent initiation of transcriptional dysregulation.

### 2.4 scRNAseq shows cell type‐specific transcriptome changes with age

To gain further insight into the age‐associated changes in transcriptome expression at the level of individual cell types, we performed differential expression analysis across the ageing time points within the major retinal cell types. We utilized the regression modelling in Monocle3 to determine transcripts that changed with age (with cut‐off: *q*‐value <1E‐5). While single‐cell differential analysis can have high false‐positive rates (Squair et al., 2021), we noted interesting cell type‐specific trends within the scRNAseq dataset (Table S3). The changes observed below are between 6‐ and 18‐week‐old animals.

For example, we noted a decrease in transcript levels of the *atp1b3b* gene in retinal ganglion cells (log_2_FC = −1.008, *q*‐value = 3,01E‐13). This gene encodes the sodium/potassium‐transporting ATPase subunit beta family b enzyme, which is essential in maintaining the electrochemical gradient across the cell membrane. Although ATP1B3 has not been directly linked to glaucoma, dysregulation of ion channel activity is known to disrupt ion balance within the retinal ganglion cells (Boal et al., 2023; Chen et al., 2013), leading to oxidative stress and apoptosis, both present in glaucomatous eyes (Ma et al., 2022). The decreased *atp1b3b* expression observed in aged killifish ganglion cells might thus indicate loss of cellular homeostasis and dysregulation of ion channel activity in retinal ganglion cells.

Interestingly, glial (Müller glia and oligodendrocytes) and immune cell populations exhibited similar transcriptional responses to age‐related stress. We observed increased transcript expression of *fkbp5* in Müller glia (log_2_FC = 1.257, *q*‐value = 9.75E‐80), immune cells (log_2_FC = 1.083, *q*‐value = 1.44E‐7), and oligodendrocytes (log_2_FC = 1.160, *q*‐value = 2.99E‐10). FKBP5 is an important modulator of stress responses through its binding to heat‐shock protein 90 (HSP90), and increased levels of FKBP5 have been described to lead to a prolonged stress response (Zannas et al., 2016). Notably, Müller glia also showed increased levels of *hsp90aa1.2* (log_2_FC = 0.497, *q*‐value = 1.07E‐8), another gene linked to stress responses (Zannas et al., 2016). As FKBP5 is an important co‐chaperone of the glucocorticoid receptor signalling pathway (Binder, 2009), and the subsequent stress response management, we also evaluated another regulatory player involved within this pathway, namely KLF9 (Gans et al., 2020). Interestingly, like in zebrafish (Gans et al., 2021), *klf9* showed synchronous upregulation (log_2_FC = 1.249, *q*‐value = 1.03E‐42) with *fkbp5* in aged killifish Müller glia.

Together, these data indicate that ageing‐associated transcriptional dysregulation occurs across all retinal cell types. We observe that numerous genes that function as stress response genes within glial and immune cell populations display upregulated expression, suggesting an influence for these cell types and transcripts for homeostatic management of stress responses or direct contributions to ageing phenotypes.

## 3 DISCUSSION

The aim of this study was to identify the molecular changes in the ageing killifish retina that underlie retinal ageing/disease phenotypes by utilizing transcriptomics. We observed age‐related transcriptome changes between 6‐, 12‐, and 18‐week‐old female killifish retinas. Male killifish show a distinct age trajectory compared to females, experiencing higher mortality rates earlier in life (Reichard et al., 2022). Therefore, male killifish were not included in this study to eliminate sex‐related variance within a single age group. However, by doing so, transcriptional differences resulting from biological sex during ageing cannot be addressed. Indeed, as African turquoise killifish possess a heteromorphic XY sex chromosome system, with males being heterogametic (Reichard et al., 2022; Štundlová et al., 2022), Y‐bound genes were not considered in this study. Therefore, comparing aged male and female killifish in future studies may be of interest. Our investigations revealed that the ageing killifish retina has dysregulation in pathways associated with oxidative stress, gliosis, and inflammageing, which were previously reported as hallmarks of brain ageing (Azam et al., 2021; Mattson & Arumugam, 2018). Additionally, increases in oxidative stress and inflammatory responses were corroborated by comparison to the ageing mouse retina, which had enrichment of similar cellular pathways (Xu et al., 2022). Interestingly, a previously published transcriptome study on the human and macaque retina also reported an age‐related increase in oxidative stress and inflammation (Yi et al., 2021). In contrast to the aged murine retina (Xu et al., 2022) and a previous histological study of ageing killifish retina (Vanhunsel et al., 2021), we did not observe increased senescence marker gene expression in the killifish retina at 18 weeks of age by bulk RNAseq. This may be because previous killifish studies showed high levels of senescence‐associated beta‐galactosidase signal in the RPE on histological preparations of 18‐week‐old retinas (Vanhunsel et al., 2021), a cell type that was removed during dissection in this study to focus on the neural retina. Further studies are required to understand the implications of senescence‐related pathways and other potential important age‐related changes of essential processes in RPE as it contributes to certain retinal diseases, such as AMD (Somasundaran et al., 2020). Notably, minor increases in beta‐galactosidase signal were previously detected in the neural retina of 24‐week‐old fish (Vanhunsel et al., 2021), suggesting the transcriptional dysregulation identified in our study may precede senescence‐associated phenotypes. Overall, determining the impact of genetic or pharmacologic manipulation of age‐associated senescence, oxidative stress, and/or inflammation signatures on retinal health and function may open avenues for novel targets amenable for therapeutic intervention. Pharmacological manipulation of senescence using senolytic drugs has recently been shown to remove senescent cells from the aged killifish telencephalon, thereby restoring the neurogenic potential of the aged killifish brain (Van houcke et al., 2023). Similar pharmacological approaches targeting the identified ageing processes might thus have the potential to rejuvenate the aged retina.

We also observed transcriptional changes of several genes associated with age‐related human retinal disease, which suggests that the killifish is a valuable translational model. Indeed, bulk RNAseq revealed changes in specific genes, such as *optn*, *htra1b*, *and pcdh15a*, associated with glaucoma, AMD, and Usher syndrome, respectively (Ahmed et al., 2001; Rezaie et al., 2002; Yang et al., 2006). As increased levels of *optn* have been reported as neuroprotective (Markovinovic et al., 2018; Weil et al., 2018), the observed rise in *optn* expression in the aged killifish retina may suggest a compensatory phenotype attempting to attenuate age‐related cellular stress. Altogether, our data highlight the value of old killifish retina as a model to study pathomechanisms of several human ageing retinal diseases. The killifish retina also has the potential for investigating age‐related neurodegenerative brain pathologies (Bergmans, Raes, et al., 2023; de Bakker & Valenzano, 2023). For example, we observed that expression of the Parkinson‐associated gene, *snca*, increases with age. Previous studies reported a similar increase in the aged killifish brain that likely contributes to Parkinsonian‐like phenotypes, such as protein aggregation and Lewy body formation (Bagnoli et al., 2022; Matsui et al., 2019). As the killifish is genetically amenable to transgenesis and CRISPR/Cas9 mutagenesis (Bedbrook et al., 2023; Hartmann & Englert, 2012; Rozenberg et al., 2023), it will be invaluable to perform genetic manipulation of disease genes within this rapidly ageing vertebrate.

Single‐cell transcriptomics were used to delineate the cellular landscape of the killifish retina. The killifish retina contains all major cell classes, as well as oligodendrocytes, previously reported to be present in fish and avian retinas (Nakazawa et al., 1993; Santos‐Ledo et al., 2023). Differential gene expression analysis of the scRNAseq dataset revealed cell type‐specific transcriptomic changes. Notably, there was an upregulation of glucocorticoid receptor‐mediated stress response, evidenced by increased expression levels of both *fkbp5* and *klf9* in aged killifish Müller glia. FKBP5 and KLF9 serve as feedback systems for glucocorticoid receptor signalling, ultimately leading to a reduced receptor sensitivity (Gans et al., 2020, 2021; Zimmer et al., 2020). Their heightened expression levels in the aged killifish retina may indicate a chronic exposure to glucocorticoids. This observation aligns with the glucocorticoid hypothesis of brain ageing, which posits that prolonged exposure to glucocorticoids accelerates the ageing process in the brain (Landfield et al., 2007). Furthermore, elevated KLF9 levels have been reported to be involved in maintaining the quiescent state of radial‐glial neural stem cells in the hippocampus of mice (Guo et al., 2022). Consequently, it could be speculated that aged killifish Müller glia also transition into a quiescent state, potentially negatively impacting Müller glia‐derived neurogenesis and tissue repair.

Additionally, age‐related changes in gene expression across the cell types were determined by combining the single‐cell with the bulk RNAseq datasets. In general, we observed age‐related transcriptomic dysregulation within all retinal cell populations, showing that differentially expressed genes from bulk RNAseq experiments displayed increased expression across all cell types upon ageing. Similar age‐related transcriptional dysregulation was described in *C. elegans* using a full organism single‐cell atlas for a confined number of genes, although the mechanisms contributing to this altered gene expression remain elusive (Roux et al., 2023). One potential mechanism behind the changes in gene expression across various cell types includes a modified epigenome. Previous studies examining the retinas and RPE of AMD patients reported global changes in chromatin accessibility (Wang et al., 2018). It will be interesting to determine the extent to which age‐related transcriptomic changes in the killifish retina are mediated by alterations to the epigenetic landscape. Furthermore, it is important to note that RNA levels are not indicative of protein levels in vivo. Decoupling of transcription and protein synthesis has been observed in killifish and was reported to worsen with age (Kelmer Sacramento et al., 2020). As such, the transcriptional changes reported here may not be fully reflected at the protein level.

In summary, we have identified age‐related transcriptional dysregulation in the killifish retina. This work highlights that the rapidly ageing killifish can be utilised to define the molecular features underlying age‐related retinal pathology and vision loss. As the retina is considered a window to the brain, our data may provide insight into processes occurring within the brain Killifish can be used to study the consequence of molecular dysregulation on CNS health and identify means to prevent disease.

## 4 MATERIALS AND METHODS

### 4.1 Fish husbandry

Given the variation in ageing rates, exclusively young adult (6‐week‐old), middle‐aged (12‐week‐old), and old (18‐week‐old) female GRZ‐AD inbred strain African turquoise killifish were used in this study. The three age groups were selected based on an in‐house survival curve and a thorough characterization of the ageing phenotype within the killifish visual system (Van houcke et al., 2021; Vanhunsel et al., 2021). Fish were bred, grown, and maintained in‐house as described (Bergmans, Serneels, et al., 2023; Van houcke et al., 2021; Vanhunsel et al., 2022, 2021). The Ethical Committee for Animal Experimentation of KU Leuven, strictly following the European Communities Council Directive of 2010 (2010/63/EU) and Belgian legislation (Royal degree of 29 May 2013), approved all animal experiments.

### 4.2 Tissue collection/processing

#### 4.2.1 Sample collection and dissociation for (sc)RNAseq

Fish were euthanized using 0.1% tris buffered tricaine (Merck) in system water. Retinas were collected as previously described (Van Dyck et al., 2023). Briefly, the dermal and scleral layer of the cornea, and the lens were removed, exposing the retina. The retinal pigment epithelial was rinsed off with sterile Dulbecco PBS (Thermo). Retinas were collected and either snap frozen in liquid nitrogen for bulk RNAseq or collected in single‐cell suspension medium for scRNAseq.

For bulk RNAseq, retinas of one fish were pooled as a single sample and 10 samples used per group. Samples were digested using Tri‐reagent (Sigma‐Aldrich) and RNA purified with RNeasy kit (Qiagen) (Bergmans, Vanhunsel, Zandecki, et al., 2023). RNA quality and integrity were assayed using the DNA 12000 kit on the Bioanalyzer (Agilent) at the KU Leuven Genomics Core (https://www.genomicscore.be/).

For scRNAseq, single‐cell suspensions were generated as described (Bergmans, Serneels, et al., 2023). Briefly, retinas from either three (6‐week‐old) or two (12‐ or 18‐week‐old) fish were pooled and collected in complete Leibovitz's 15 (cL15) medium. Retinas were rinsed 3× with cL15 before digestion using sterile activated papain (16 U/mL, Worthington) for 30 min at 28°C. Digested retinas were rinsed 3× with cL15. Cell suspensions were obtained after mechanical trituration (10× with both a p1000 and a p200 pipet) in sterile PBS with 1% bovine serum albumin (fraction V, Sigma‐Aldrich). Cell clumps were removed using a 40 μm strainer (PluriSelect). Cell viability was measured with the LUNA Automated Cell Counter at the KU Leuven Genomics Core (https://www.genomicscore.be/). Two biological samples were processed at each age, totalling six scRNAseq runs.

#### 4.2.2 Tissue collection for histology

Fish were euthanized as above. Intracardial perfusion with PBS and 4% paraformaldehyde (PFA, Merck‐Aldrich) was executed, as described (Bergmans, Vanhunsel, et al., 2023). Eyes were collected and fixed overnight in 4% PFA then rinsed 3× in PBS. Eyes were cryoprotected with increasing sucrose concentrations to 30% in PBS and embedded in 1.25% agarose and 30% sucrose in PBS for cryosectioning. 10 μm cryosections were collected on SuperFrost Plus Adhesion Slides (Epredia) and stored at −20°C until use (Bergmans, Vanhunsel, et al., 2023) .

### 4.3 RNA‐sequencing

#### 4.3.1 Bulk RNAseq analysis

Libraries were generated using the QuantSeq 3′ (Lexogen) mRNA protocol and sequenced using Illumina HiSeq 4000 (Illumina; 50 bp single end reads). On average, 8.2 M reads were obtained for each sample. Reads were trimmed using Trim‐Galore! (v0.6.7), a wrapper for Cutadapt (v3.4) and FastQC (v0.11.9). Trimmed reads were mapped to a custom‐built transcriptome (see below) using STAR (v2.7.0) (Dobin et al., 2013). Reads were cleaned using Samtools (v1.9.4), gene counts generated by HTseq (v0.11.2) (Anders et al., 2015), and visualizations generated in R (v3.6.1). Comparative analysis between individual ages was performed using EdgeR (v3.42.4) (Robinson et al., 2009). Thresholds for statistical significance were set at |log_2_FC| ≥ 1 and FDR <0.05. As the killifish reference genome in a draft phase, gene annotation for those passing statistical thresholds were checked by hand and in some instances re‐annotated to conform to the nearest ortholog using BLAST (Altschul et al., 1990). Heatmaps were generated using ComplexHeatmap (Gu et al., 2016) and PCA using the R stats package while GO was performed using the Gene Ontology knowledgebase resources (Aleksander et al., 2023; Ashburner et al., 2000).

#### 4.3.2 10× genomics sequencing and analysis

Single‐cell suspensions with a viability score greater than 85% (ranging from 87.20% to 95.00%) were loaded on to the 10× Genomics microfluidic system with a capture target of 10,000 cells per sample. scRNAseq was performed using standard protocols for 10× Genomics v3 3′ sequencing. Samples were sequenced to a target read depth of ~160 million paired end reads per library (19,291 reads/cell on average) using Illumina NovaSeq. Library prep and sequencing were performed at the KU Leuven Genomics Core (https://www.genomicscore.be/).

To increase mapping efficiency of sequencing reads, we built a custom transcriptome, utilizing the reference sequence (GRZ Assembly, 05/2015) (Valenzano et al., 2015) and custom annotations. Using GffCompare tool, the annotations were customized from Cellranger “mkgtf” by combining the NCBI annotation file (NFINdb) with the in‐house sequenced PACBIO‐ISOSEQ annotation files for killifish telencephalon. This way the gtf files from PACBIO long‐reads and the NFIN db reference transcripts were efficiently merged (Rajagopal et al., 2021). This helped us improve our final genomic annotation file and ensured higher mapping accuracy and coverage for performing single‐cell RNAseq analysis. Use of the custom transcriptome increased mapped reads by 11%, resulting in a 9% increase in cell number compared to the standard reference transcriptome alone. Sequencing reads were mapped to the custom transcriptome using CellRanger‐7.0.1.

Mapped scRNAseq data was processed in Monocle3 (monocle3_1.2.9) (Cao et al., 2019; Trapnell et al., 2014). Cells utilized for analysis contained greater than 1000 unique transcripts and less than 20,000 transcripts. UMAP dimension reduction was performed on 3327 high variance genes, determined from the transcripts/10,000 transcripts normalized count matrix, with the top 22 principal components used as input into the Monocle3 reduce_dimension function (max_components = 2, reduction_method = “UMAP”, preprocess_method = “PCA”, umap.metric = “euclidean”, umap.min_dist = 0.25, umap.n_neighbors = 25, build_nn_index = TRUE). Cell type cluster assignment was performed using known markers of vertebrate retinal cell types, as performed previously (Clark et al., 2019; Lu et al., 2020). Further subclustering of individual cell types (amacrine, horizontal, cones, and bipolar cells) was performed to examine marker genes of putative subtypes.

### 4.4 IHC, in situ HCR, and imaging

For IHC, slides were warmed to room temperature (RT) and rehydrated with PBS. Antigen retrieval was performed by boiling in sodium citrate pH 6.0 for 20 min. Slides were washed with PBS and blocked (10% normal goat serum, 1% BSA) for 1 h. Primary antibody mixed in blocking solution was applied to slides overnight at 4°C. Antibody was washed 3× with PBS, then incubated overnight in secondary anitbody (1:1000) at 4°C. After rinsing with PBS 3×, slides were mounted and imaged. See Table S4 for antibody list.

Fluorescent in situ HCR v3.0 was performed as described (Choi et al., 2018; Elagoz et al., 2022; Van houcke et al., 2021). See Table S5 for probe information. Briefly, probes were obtained using Easy_HCR (https://gitlab.com/NCDRlab/easy_hcr) and ordered at Integrated DNA Technologies (IDT). Cryosections were dried at 37°C, rehydrated using diethyl pyrocarbonate (DEPC, Acros)‐treated PBS (0.1% v/v), and sections permeabilized using proteinase K (10 μg/mL in PBS‐DEPC, Roche) for 10 min at 37°C. Permeabilization was stopped by incubation in 4% PFA for 10 min at RT. Slides were rinsed 3× before incubation in hybridisation buffer (formamide, 30%; NaCl, 0.75 M; sodium citrate, 75 mM; citric acid pH 6.0, 9 mM; Tween20, 0.1%; Heparin, 50 μg/mL; Denhardt's solution 50×, 2%; Dextran, 10%) for 30 min at 37°C. Probes were added at a final concentration of 6.67 nM in probe hybridization buffer and hybridization was accomplished overnight at 37°C. Slides were rinsed 3× and incubated with amplification buffer (NaCl, 0.75 M; sodium citrate, 75 mM; Tween20, 0.1%; Dextran, 10%) for 30 min at RT. Both hairpins to visualize a probe set were prepared separately: 3 pmol of each hairpin (H1 and H2) was incubated for 90 s at 95°C and cooled down to RT for 30 min. Hairpins were added to the slides at a final concentration of 40 nM in amplification buffer and incubated overnight at RT. Nuclei were stained with DAPI and tissue mounted with Mowiol®.

Slides were imaged on a Zeiss LSM900 microscope with a Plan‐Apochromat 20X 0.8 NA objective in Airyscan CO‐2Y mode. Images were acquired as mosaic Z‐stacks covering the whole thickness of the section and visualized as maximum projections.

## AUTHOR CONTRIBUTIONS

Conceptualization and experimental design: SB, NCLN, LuM, LA, PAR, RBM, BSC, and LM. Data curation: SB, NCLN, AMK, JDDS, and RBM. Bioinformatic analysis: EH, C‐KH, PAR, and BSC. Reference genome: RA and LA. Visualization: SB, NCLN, PAR, and BSC. Writing—original draft preparation: SB and NCLN. Reviewing and editing: SB, NCLN, LuM, EH, AMK, JDDS, RA, C‐KH, LA, PAR, RBM, BSC, and LM. Funding acquisition: SB, NCLN, LuM, RBM, and LM. Project administration: SB, NCLN, PAR, BSC, RBM, and LM. Supervision: PAR, BSC, RBM, and LM.

## CONFLICT OF INTEREST STATEMENT

The authors declare that they have read and approved the manuscript and have no conflicts of interest.

## Supporting information


Figure S1.

Figure S2.

Figure S3.

Figure S4.

Figure S5.

Figure S6.

Figure S7.

Figure S8.

Figure S9.

Table S1.

Table S2.

Table S3.

Table S4.

Table S5.

## Data Availability

Raw and processed bulk RNA and scRNAseq data are available for download through GEO (GSE255363 and GSE255364, respectively).
