# Supplementary material for: Age‐related dysregulation of the retinal transcriptome in African turquoise killifish
Source: Aging Cell. 2024 May 14;23(8):e14192. doi: 10.1111/acel.14192 (PMC11320354; doi:10.1111/acel.14192)
Supplement: Supplementary file 1 — Figure S1. [file ACEL-23-e14192-s008.zip › Figure S1.docx]

Figure S1. Heatmap showing differential transcript expression across all bulk RNAseq samples. Differentially expressed genes were identified using the following thresholding criteria: FDR <0.05 and |log2FC| ≥ 1. Over age, biological replicates are highly consistent, observed changes are gradual with time, and killifish show more up-regulated genes compared to down-regulated genes. w = weeks.
