# Supplementary figures and images for: Age‐related dysregulation of the retinal transcriptome in African turquoise killifish
Source: Aging Cell. 2024 May 14;23(8):e14192. doi: 10.1111/acel.14192 (PMC11320354; doi:10.1111/acel.14192)

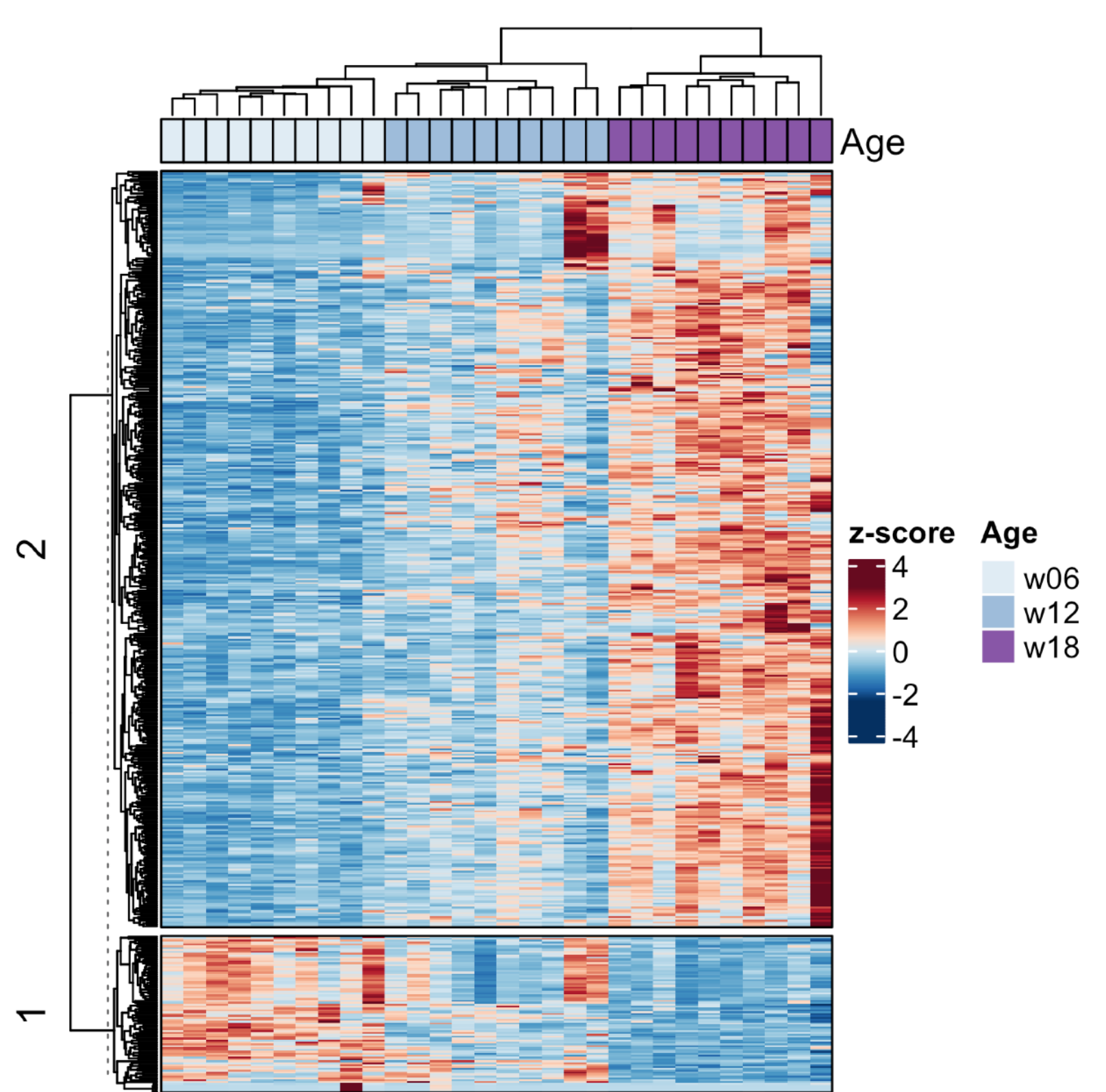

Supplement: Supplementary file 1 — Figure S1. [file ACEL-23-e14192-s008.zip › acel14192-sup-0001-Figure S1.pdf]

**A***in situ* HCR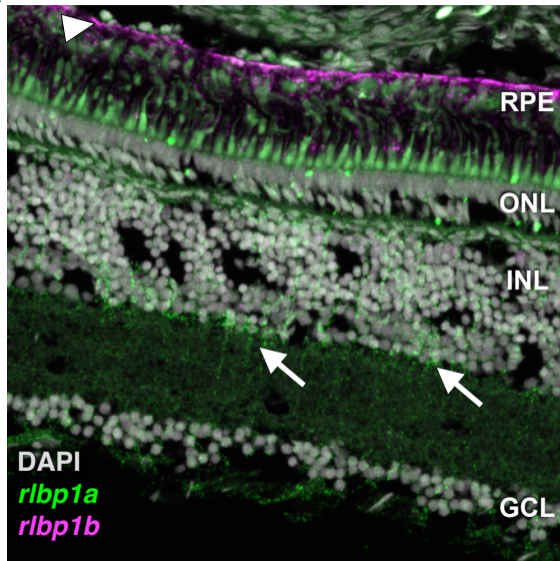**B**

IHC

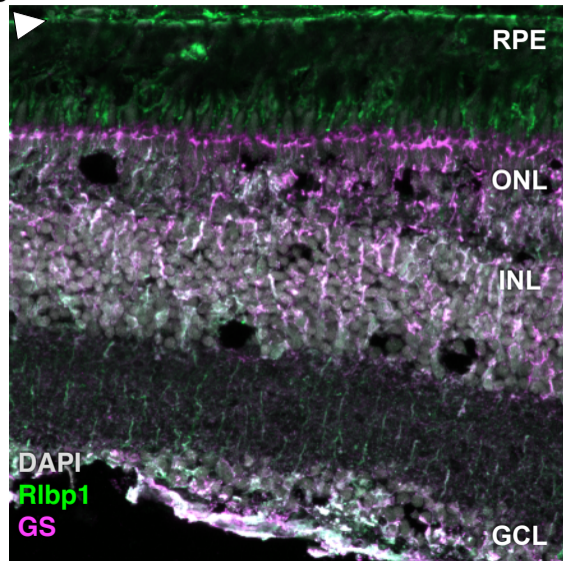

Supplement: Supplementary file 2 — Figure S2. [file ACEL-23-e14192-s012.zip › acel14192-sup-0002-Figure S2.pdf]

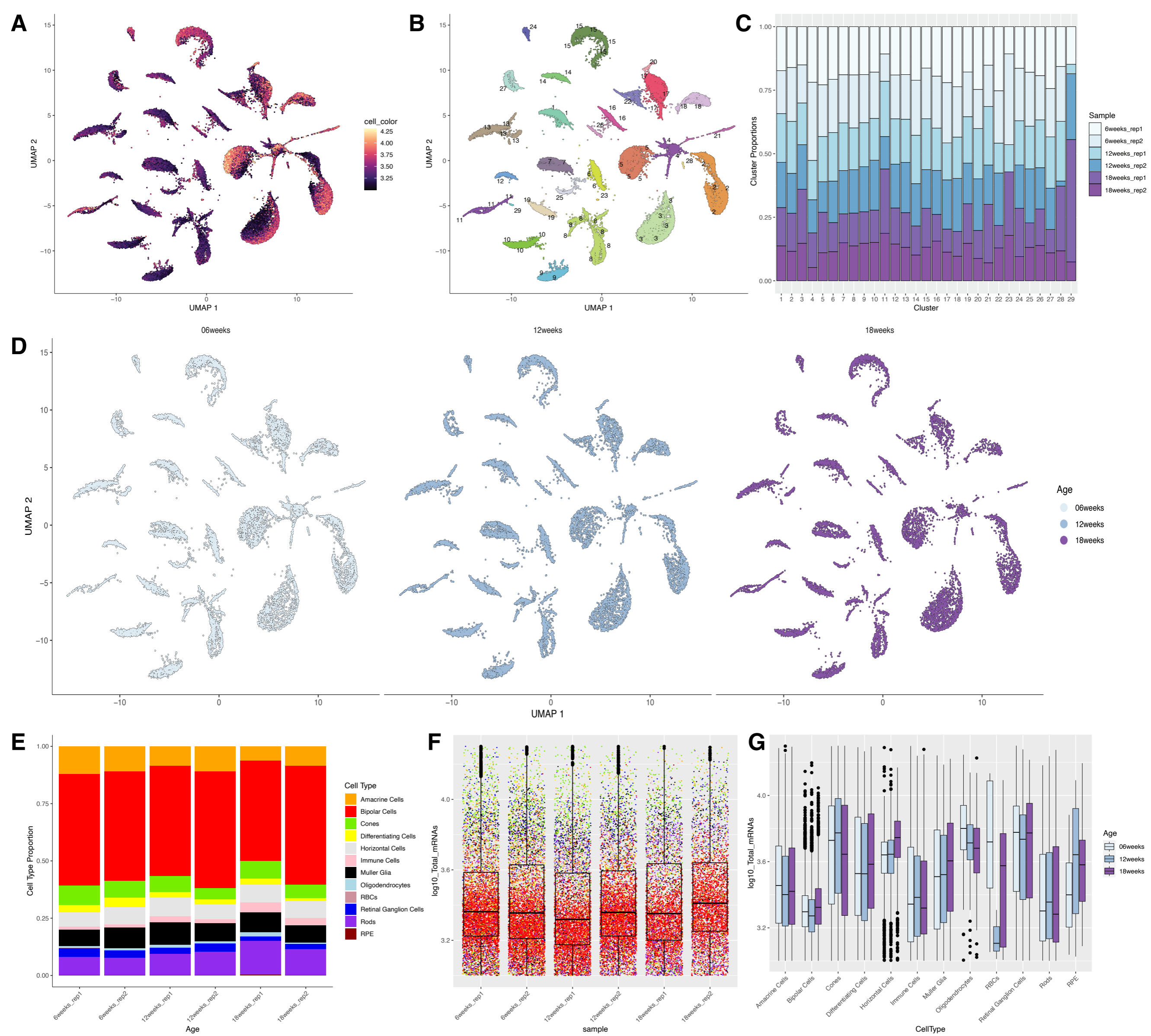

Supplement: Supplementary file 3 — Figure S3. [file ACEL-23-e14192-s010.zip › acel14192-sup-0003-Figure S3.pdf]

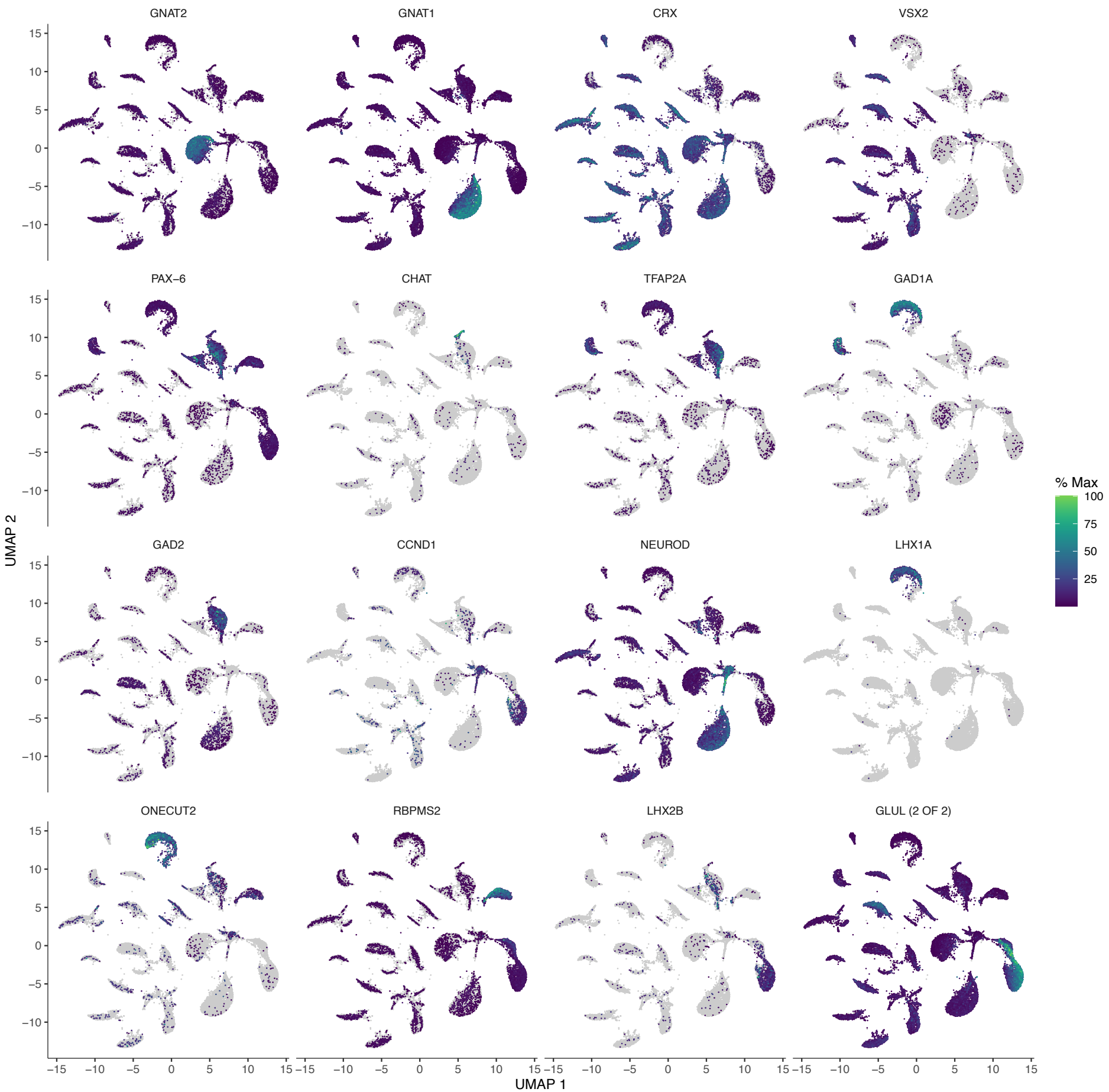

Supplement: Supplementary file 4 — Figure S4. [file ACEL-23-e14192-s007.zip › acel14192-sup-0004-Figure S4.pdf]

**A**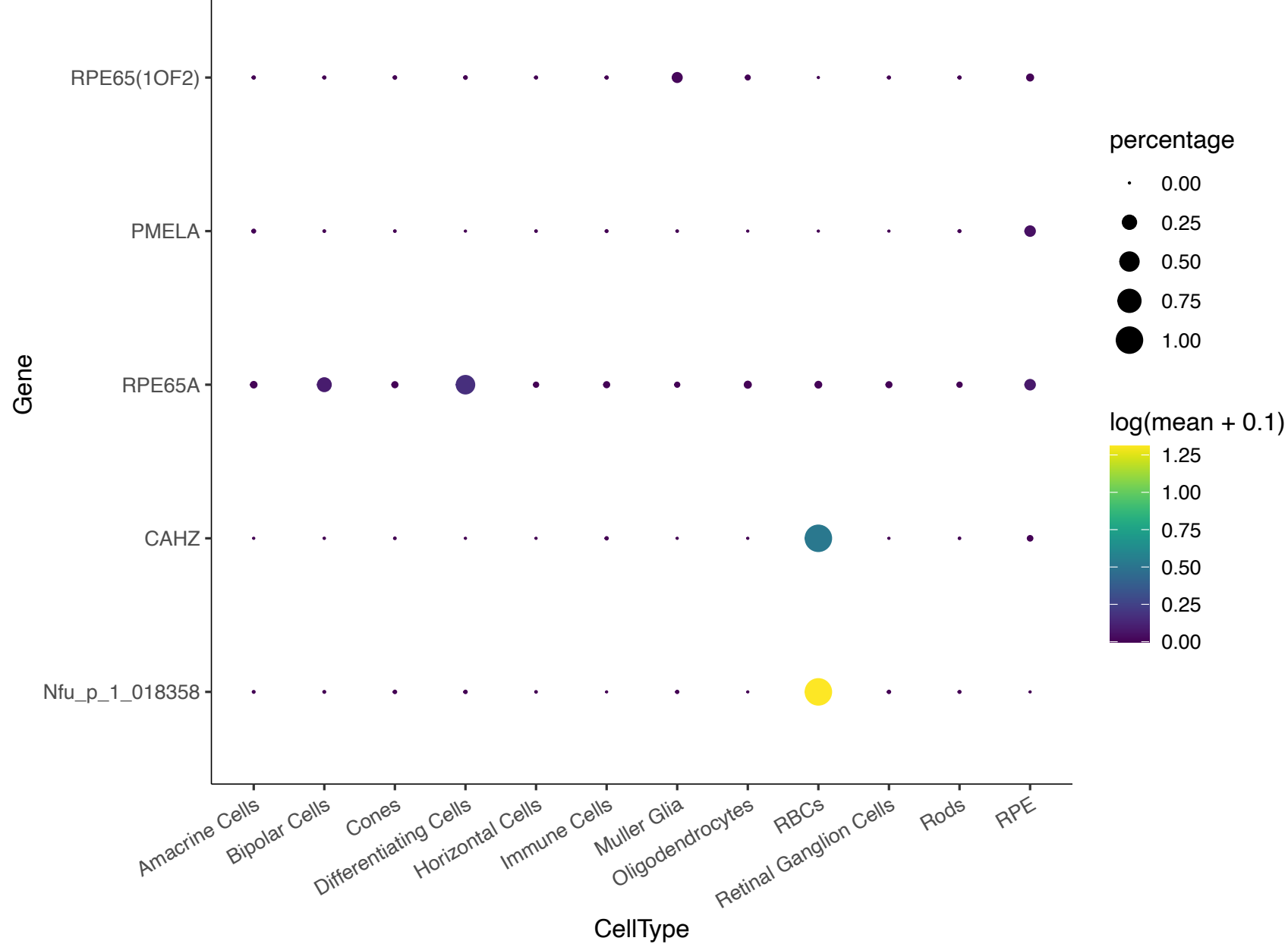**B**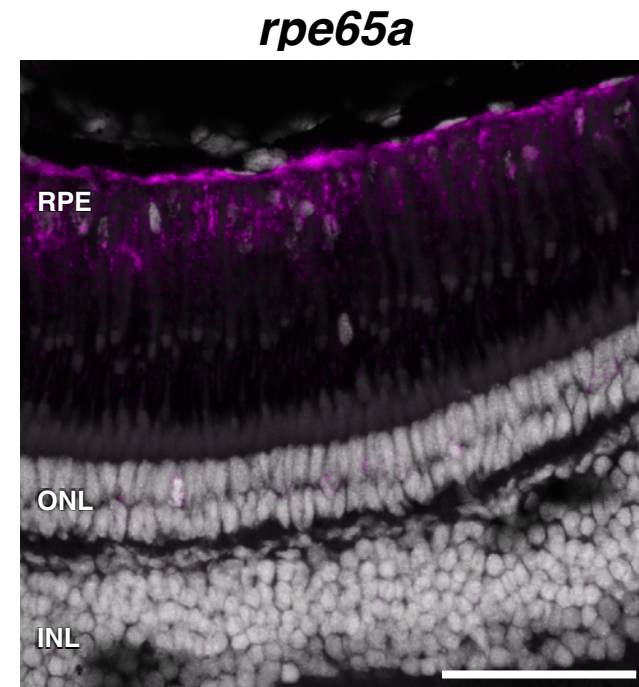

Supplement: Supplementary file 5 — Figure S5. [file ACEL-23-e14192-s006.zip › acel14192-sup-0005-Figure S5.pdf]

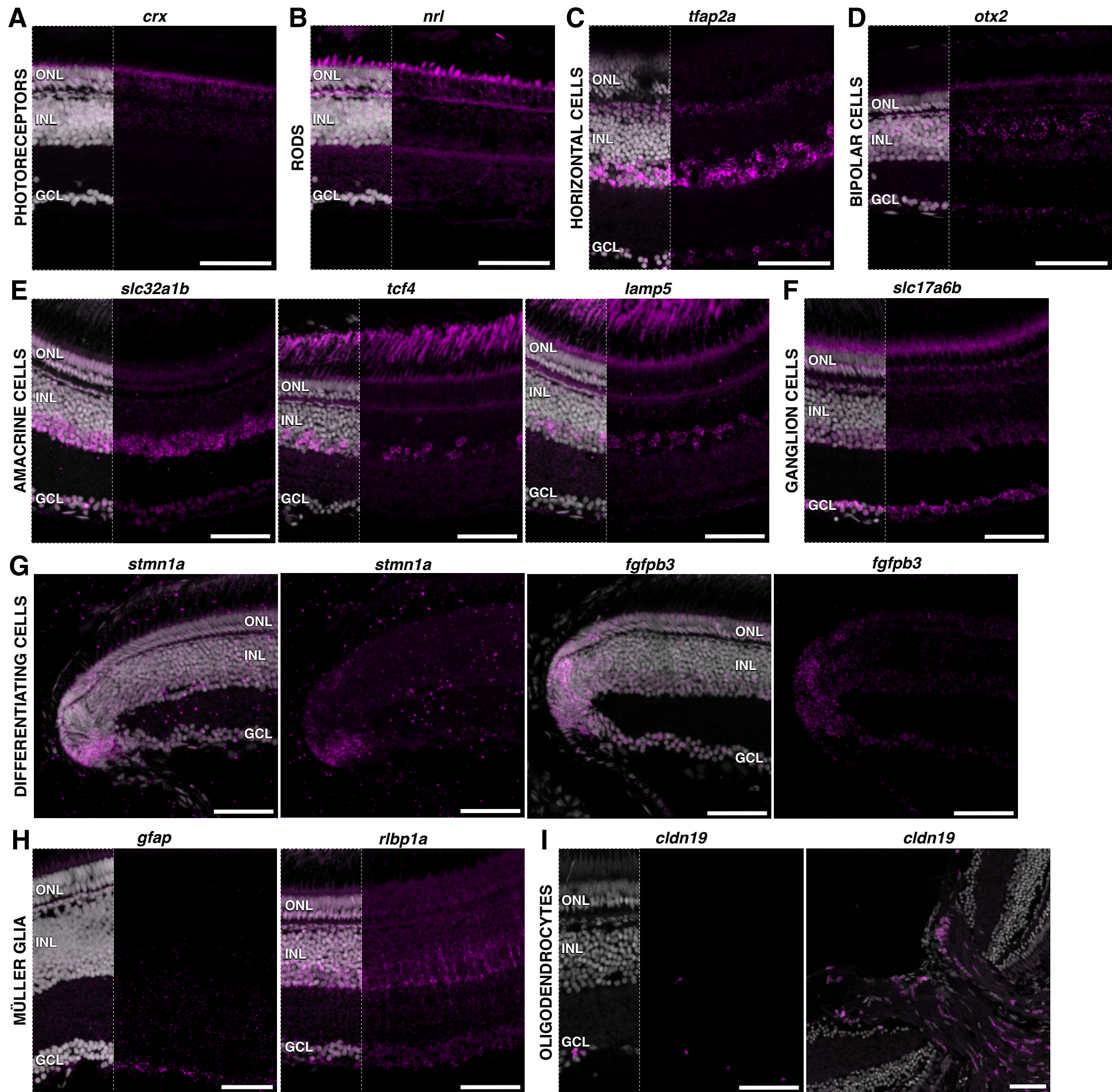

Supplement: Supplementary file 6 — Figure S6. [file ACEL-23-e14192-s004.zip › acel14192-sup-0006-Figure S6.pdf]

**A****6 vs 12**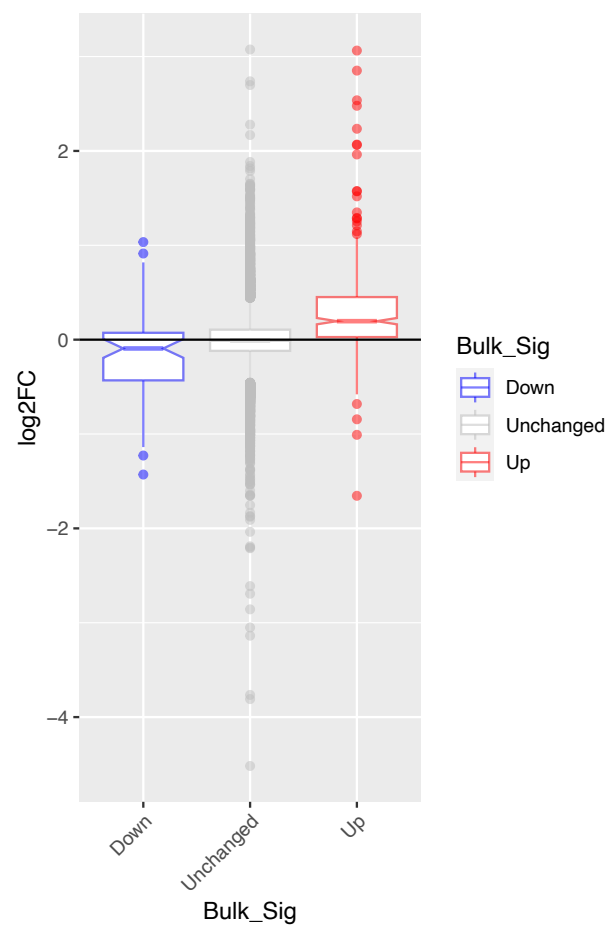**B****6 vs 12**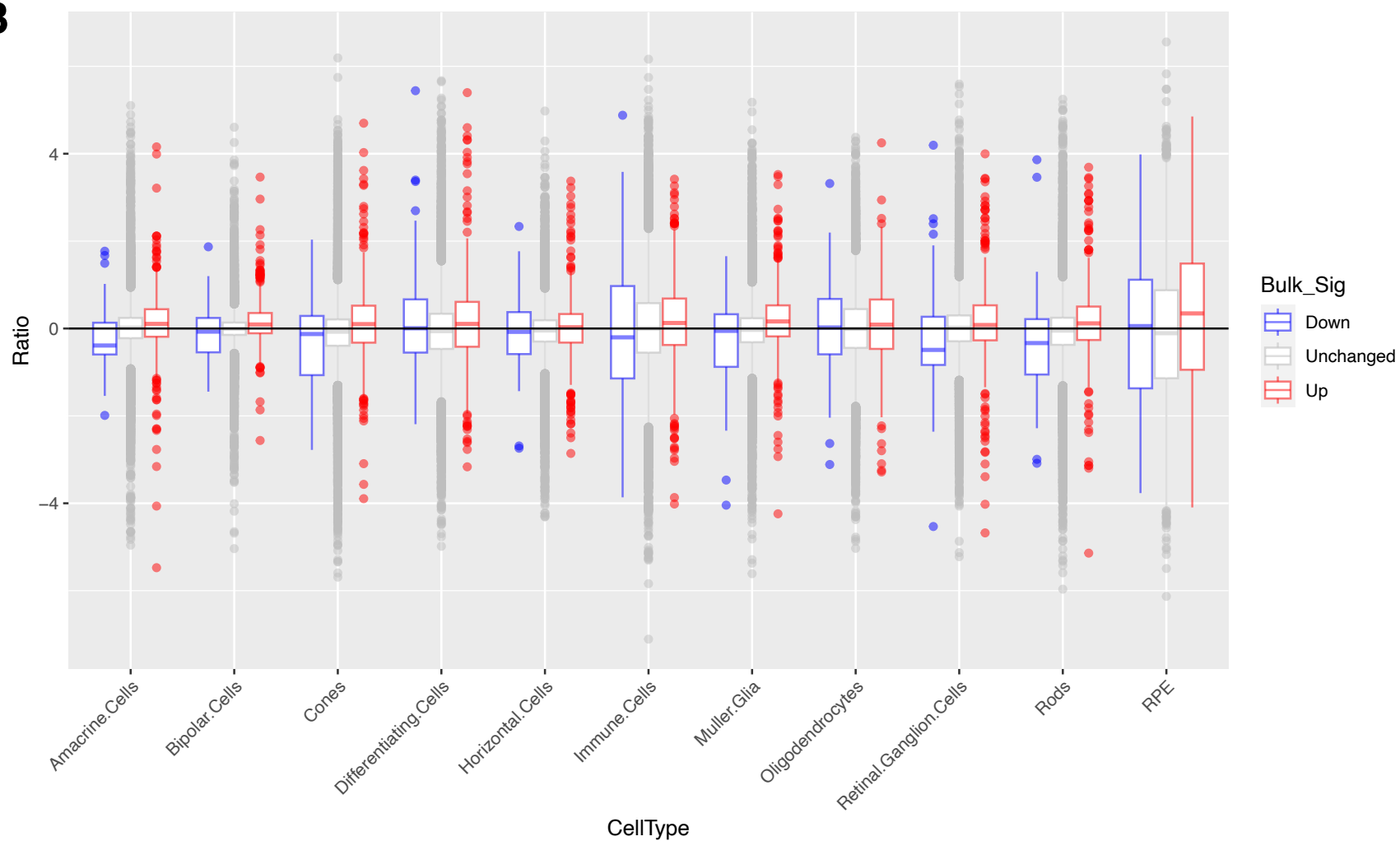**C****6 vs 18**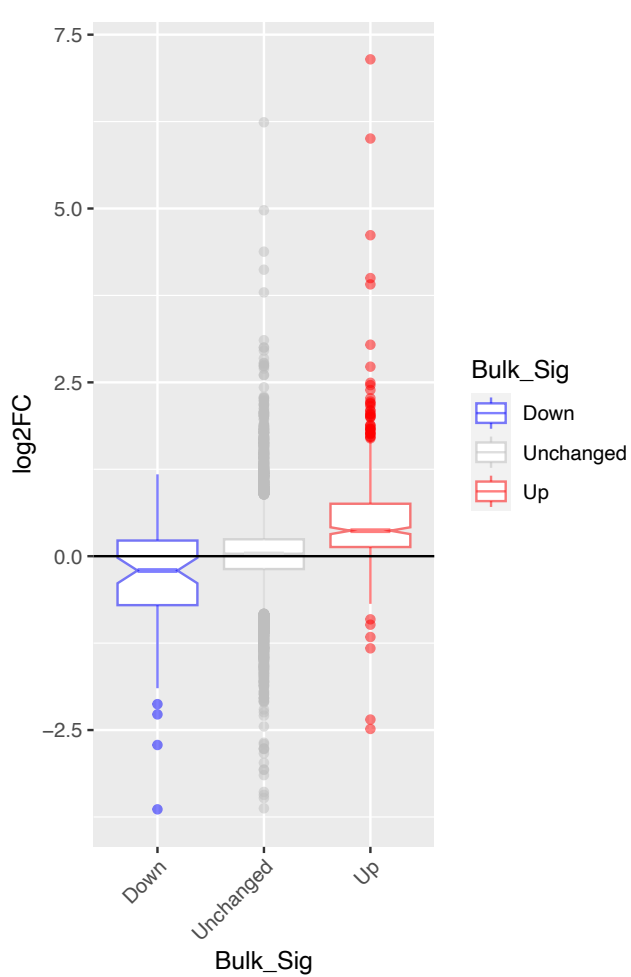**D****6 vs 18**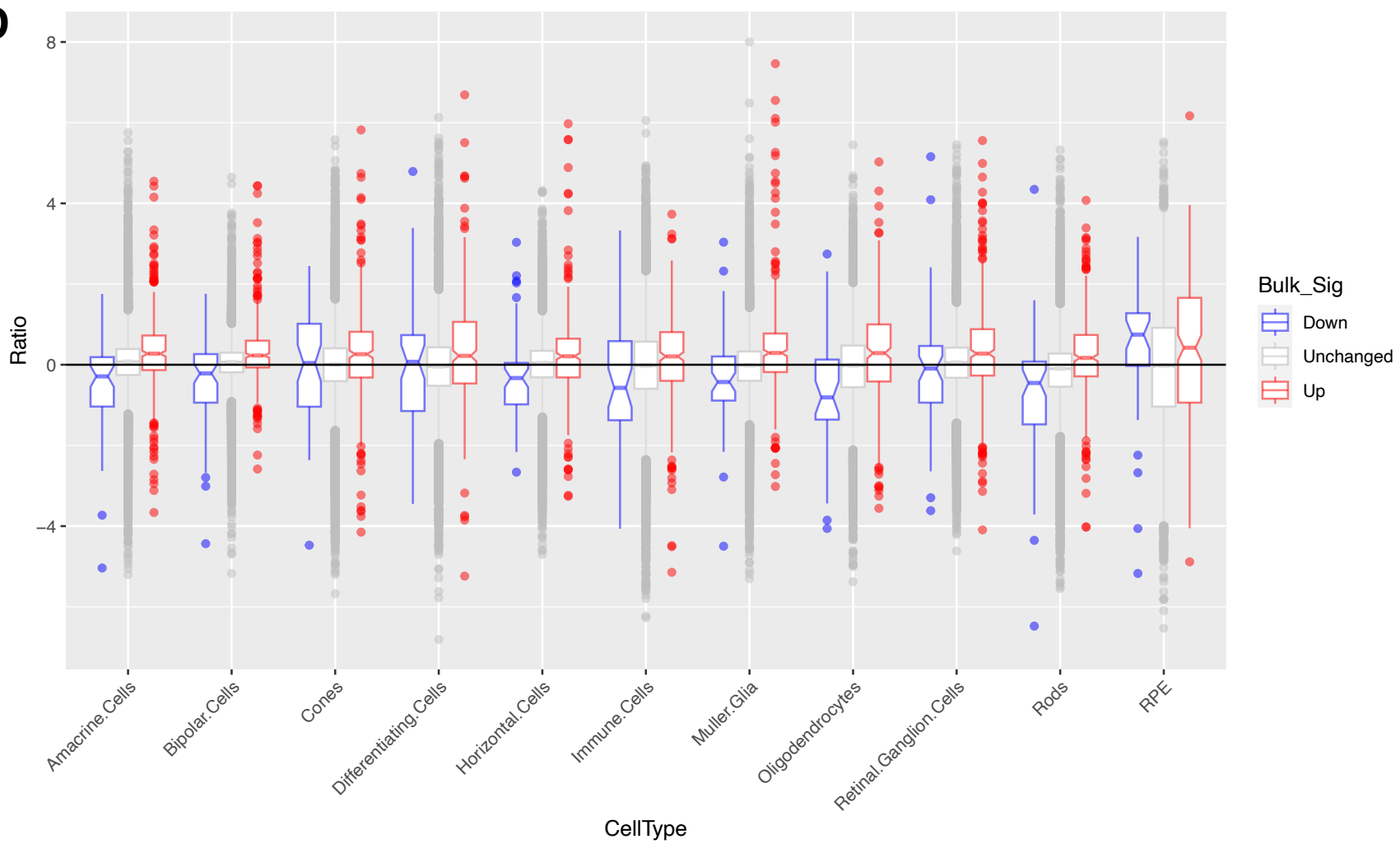

Supplement: Supplementary file 8 — Figure S8. [file ACEL-23-e14192-s011.zip › acel14192-sup-0008-Figure S8.pdf]

**A**

1

2

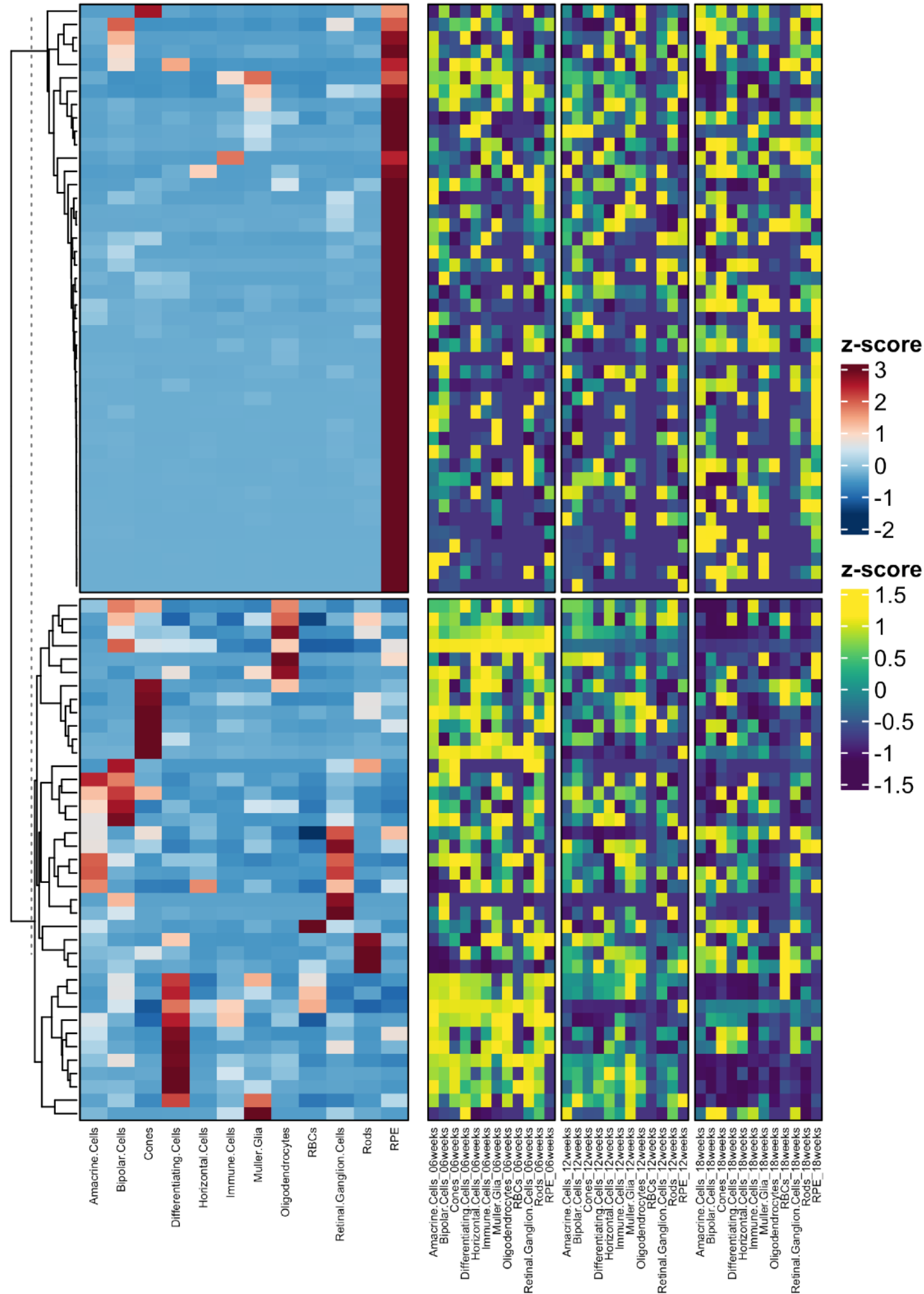**B**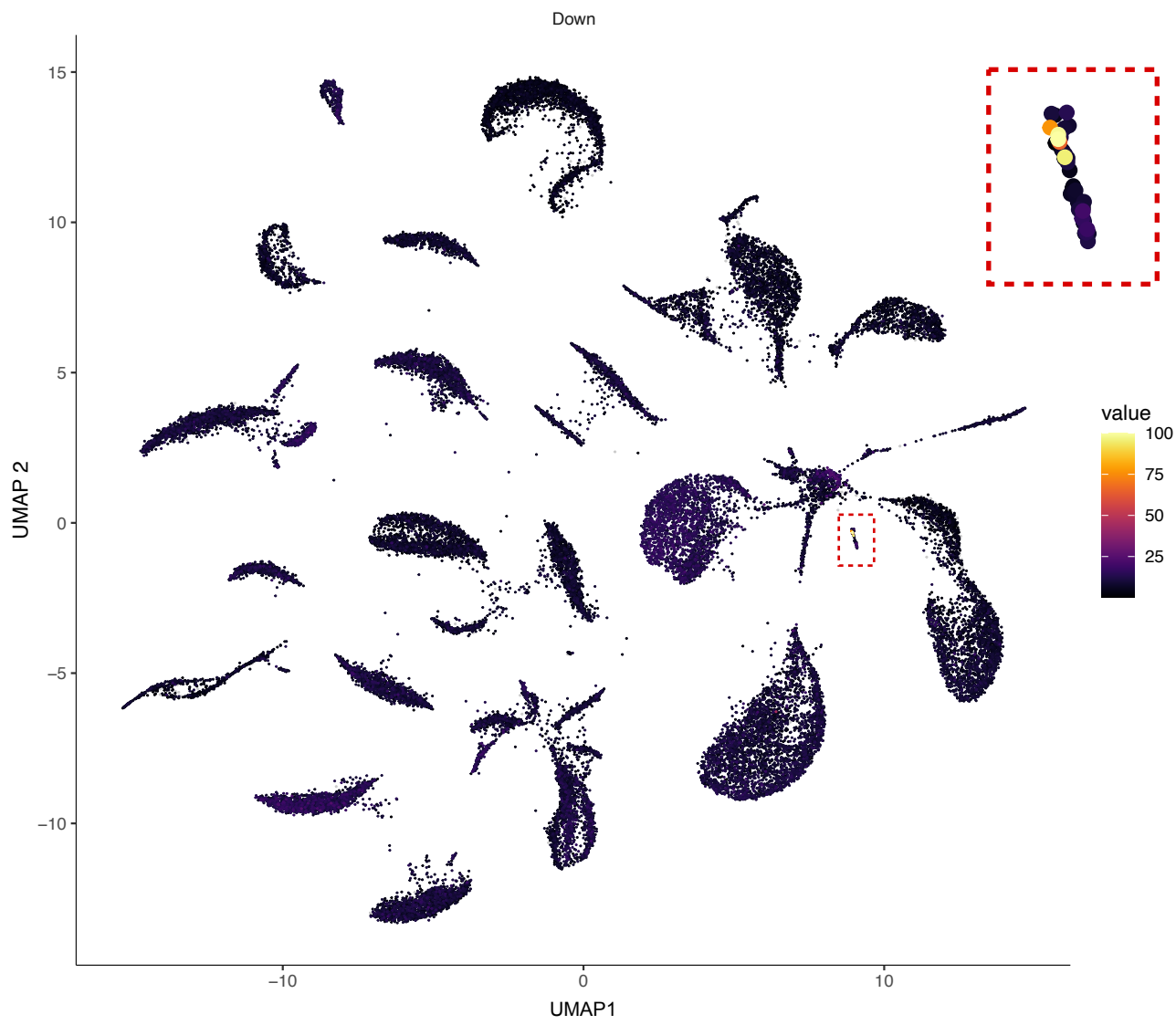

Supplement: Supplementary file 9 — Figure S9. [file ACEL-23-e14192-s009.zip › acel14192-sup-0009-Figure S9.pdf]
