# Supplementary material for: Age‐related dysregulation of the retinal transcriptome in African turquoise killifish
Source: Aging Cell. 2024 May 14;23(8):e14192. doi: 10.1111/acel.14192 (PMC11320354; doi:10.1111/acel.14192)
Supplement: Supplementary file 2 — Figure S2. [file ACEL-23-e14192-s012.zip › Figure S2.docx]

Figure S2. Rlbp1 is a Müller glia marker in the killifish neural retina. (A) rlbp1a (green) transcript is found within the neural retina, while rlbp1b (magenta) is expressed within the RPE. (B) Co-labelling of Rlbp1 and GS shows that Rlbp1 is a Müller glia marker within the neural retina via binding of Rlbp1a, although the antibody does also appear to label Rlbp1b in the RPE.
