# Supplementary material for: Age‐related dysregulation of the retinal transcriptome in African turquoise killifish
Source: Aging Cell. 2024 May 14;23(8):e14192. doi: 10.1111/acel.14192 (PMC11320354; doi:10.1111/acel.14192)
Supplement: Supplementary file 3 — Figure S3. [file ACEL-23-e14192-s010.zip › Figure S3.docx]

Figure S3. Quality control of single-cell RNA-sequencing. UMAP dimension reductions indicating the (A) log10(Total_mRNAs) or (B) cluster designation for each cell. (C) Graph depicting the sample proportions for cells within each cluster. (D) UMAP dimension reductions highlighting the presence of cells from each age (6, 12, and 18 weeks) within all clusters. (E) Cell type proportions of retinal cells within each sample. (F) Boxplot showing the average transcript count across cells within each sample. Overlayed dots show individual transcript counts for each cell, with cells coloured by the annotated cell type. (G) Boxplots highlighting the transcript counts of cell types across the profiled ages, highlighting consistency of transcript counts within cell types across the ages, but variability across individual cell types. RBC = red blood cell; RPE = retinal pigment epithelium.
