# Supplementary material for: Age‐related dysregulation of the retinal transcriptome in African turquoise killifish
Source: Aging Cell. 2024 May 14;23(8):e14192. doi: 10.1111/acel.14192 (PMC11320354; doi:10.1111/acel.14192)
Supplement: Supplementary file 4 — Figure S4. [file ACEL-23-e14192-s007.zip › Figure S4.docx]

Figure S4. Cell type marker gene expression. UMAP dimension reductions highlighting marker gene expression used for cell type calling across the dataset.
