# Supplementary material for: Age‐related dysregulation of the retinal transcriptome in African turquoise killifish
Source: Aging Cell. 2024 May 14;23(8):e14192. doi: 10.1111/acel.14192 (PMC11320354; doi:10.1111/acel.14192)
Supplement: Supplementary file 5 — Figure S5. [file ACEL-23-e14192-s006.zip › Figure S5.docx]

Figure S5. Cell type markers for RBC and RPE cells. (A) Dot plot of marker genes used to identify the RPE and RBC populations. The dot size shows the percentage of cells expressing a specific gene while the colour indicates expression level. (B) in situ HCR for rpe65a shows that rpe65a is confined to the RPE. Images are acquired as mosaic Z-stack and are visualised as maximum projections. Scale bar = 50 μm. GCL = ganglion cell layer, HCR = hybridisation chain reaction, INL = inner nuclear layer, ONL = outer nuclear layer, RBC = red blood cell; RPE = retinal pigment epithelium.
