# Supplementary material for: Age‐related dysregulation of the retinal transcriptome in African turquoise killifish
Source: Aging Cell. 2024 May 14;23(8):e14192. doi: 10.1111/acel.14192 (PMC11320354; doi:10.1111/acel.14192)
Supplement: Supplementary file 6 — Figure S6. [file ACEL-23-e14192-s004.zip › Figure S6.docx]

Figure S6. Additional spatial validation for cell type marker genes via in situ hybridisation. Additional cell type-specific markers from scRNAseq for each retinal cell type are validated on cryosections using in situ HCR. All photoreceptors are labelled by crx (A) while rods are specifically labelled by nrl (B). Horizontal (C) and bipolars (D) cells are shown by in situ labelling for tfap2a and otx2, respectively. (E) All amacrines are labelled by the canonical amacrine marker slc32a1b, while glycinergic amacrine cells are labelled with lamp5 and tcf4. (F) Retinal ganglion cells are shown by slc17a6b labelling. (G) Differentiating cells are shown in the ciliary marginal zone at the periphery of the retina by expression of stmn1a and fgfbp3. First panel for both markers is merged with nuclei stain. Second panel is marker only. (H) Characteristic radial fibre labelling for Müller glia is accomplished by HCR for rlbp1a, while low expression of gfap is detected in Müller glia endfeet. (I) in situ HCR for cldn19 shows the sparse presence of oligodendrocytes in the GCL and INL (arrowhead), while a high density is observed in the optic nerve head. (A-F, H) Merged images with nuclei shown in left third of image. Scale bars = 50 μm. GCL = ganglion cell layer, HCR = hybridisation chain reaction, INL = inner nuclear layer, ONL = outer nuclear layer.
