# Supplementary material for: Age‐related dysregulation of the retinal transcriptome in African turquoise killifish
Source: Aging Cell. 2024 May 14;23(8):e14192. doi: 10.1111/acel.14192 (PMC11320354; doi:10.1111/acel.14192)
Supplement: Supplementary file 7 — Figure S7. [file ACEL-23-e14192-s001.zip › acel14192-sup-0007-Figure S7.pdf]

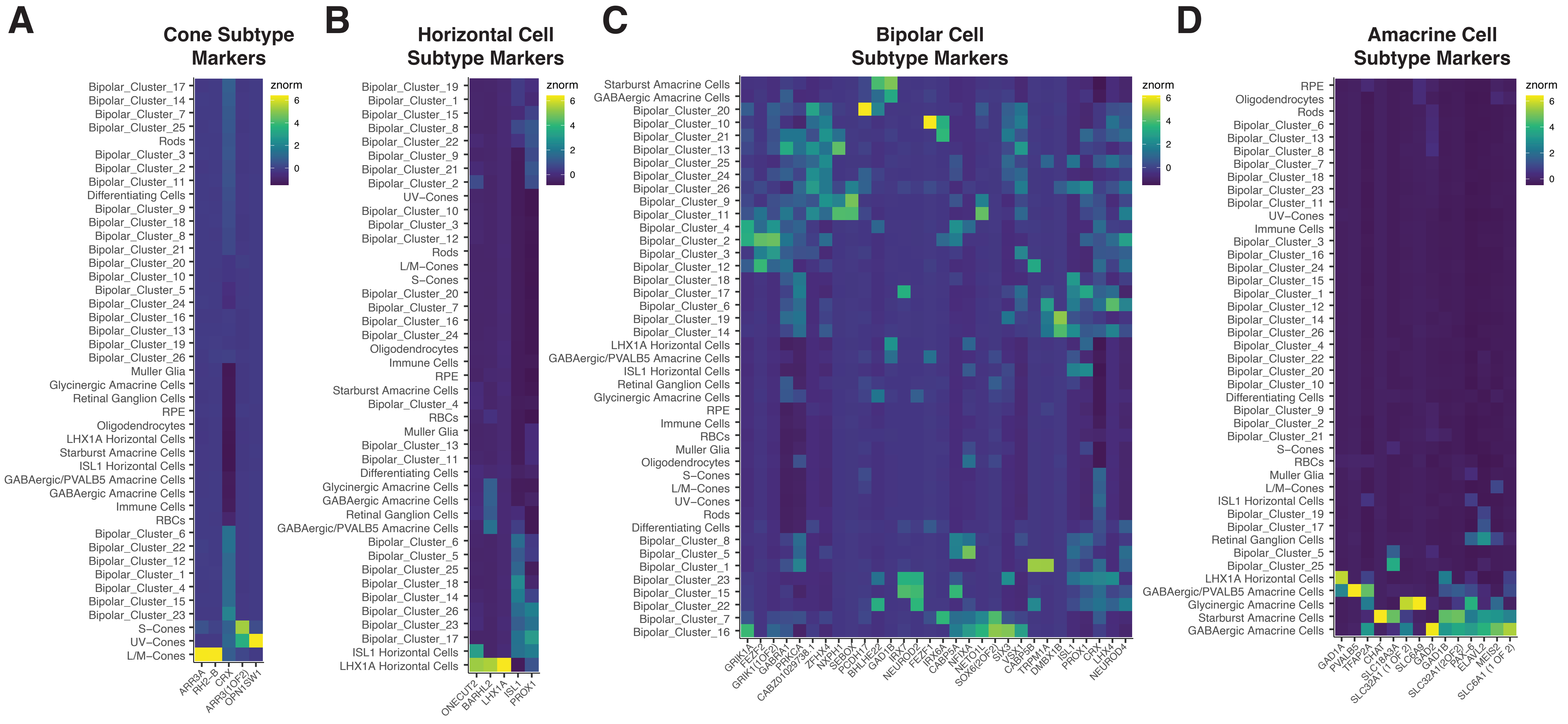
A
Cone Subtype Markers
Bipolar_Cluster_17
Bipolar_Cluster_14
Bipolar_Cluster_7
Bipolar_Cluster_25
Rods
Bipolar_Cluster_3
Bipolar_Cluster_2
Bipolar_Cluster_11
Differentiating Cells
Bipolar_Cluster_9
Bipolar_Cluster_18
Bipolar_Cluster_8
Bipolar_Cluster_21
Bipolar_Cluster_20
Bipolar_Cluster_10
Bipolar_Cluster_5
Bipolar_Cluster_24
Bipolar_Cluster_16
Bipolar_Cluster_13
Bipolar_Cluster_19
Bipolar_Cluster_26
Muller Glia
Glycinergic Amacrine Cells
Retinal Ganglion Cells
RPE
Oligodendrocytes
LHX1A Horizontal Cells
Starburst Amacrine Cells
ISL1 Horizontal Cells
GABAergic/PVALB5 Amacrine Cells
GABAergic Amacrine Cells
Immune Cells
RBCs
Bipolar_Cluster_6
Bipolar_Cluster_22
Bipolar_Cluster_12
Bipolar_Cluster_1
Bipolar_Cluster_4
Bipolar_Cluster_15
Bipolar_Cluster_23
S−Cones
UV−Cones
L/M−Cones
ARR3A
RH2-B
CRX
ARR3(1OF2)
OPN1SW1
znorm
6
4
2
0
B
Horizontal Cell Subtype Markers
Bipolar_Cluster_19
Bipolar_Cluster_1
Bipolar_Cluster_15
Bipolar_Cluster_8
Bipolar_Cluster_22
Bipolar_Cluster_9
Bipolar_Cluster_21
Bipolar_Cluster_2
UV−Cones
Bipolar_Cluster_10
Bipolar_Cluster_3
Bipolar_Cluster_12
Rods
L/M−Cones
S−Cones
Bipolar_Cluster_20
Bipolar_Cluster_7
Bipolar_Cluster_16
Bipolar_Cluster_24
Oligodendrocytes
Immune Cells
RPE
Starburst Amacrine Cells
Bipolar_Cluster_4
RBCs
Muller Glia
Bipolar_Cluster_13
Bipolar_Cluster_11
Differentiating Cells
Glycinergic Amacrine Cells
GABAergic Amacrine Cells
Retinal Ganglion Cells
GABAergic/PVALB5 Amacrine Cells
Bipolar_Cluster_6
Bipolar_Cluster_5
Bipolar_Cluster_25
Bipolar_Cluster_18
Bipolar_Cluster_14
Bipolar_Cluster_26
Bipolar_Cluster_23
Bipolar_Cluster_17
ISL1 Horizontal Cells
LHX1A Horizontal Cells
ONECUT2
BARHL2
LHX1A
ISL1
PROX1
znorm
6
4
2
0
C
Bipolar Cell Subtype Markers
Starburst Amacrine Cells
GABAergic Amacrine Cells
Bipolar_Cluster_20
Bipolar_Cluster_10
Bipolar_Cluster_21
Bipolar_Cluster_13
Bipolar_Cluster_25
Bipolar_Cluster_24
Bipolar_Cluster_26
Bipolar_Cluster_9
Bipolar_Cluster_11
Bipolar_Cluster_4
Bipolar_Cluster_2
Bipolar_Cluster_3
Bipolar_Cluster_12
Bipolar_Cluster_18
Bipolar_Cluster_17
Bipolar_Cluster_6
Bipolar_Cluster_19
Bipolar_Cluster_14
LHX1A Horizontal Cells
GABAergic/PVALB5 Amacrine Cells
ISL1 Horizontal Cells
Retinal Ganglion Cells
Glycinergic Amacrine Cells
RPE
Immune Cells
RBCs
Muller Glia
Oligodendrocytes
S−Cones
L/M−Cones
UV−Cones
Rods
Differentiating Cells
Bipolar_Cluster_8
Bipolar_Cluster_5
Bipolar_Cluster_1
Bipolar_Cluster_23
Bipolar_Cluster_15
Bipolar_Cluster_22
Bipolar_Cluster_7
Bipolar_Cluster_16
GRIK1A
FEZF2
GRIK1(1OF2)
GABRA1
PRKCA
CABZ01029738.1
ZFHX4
NXPH1
SEBOX
PCDH17
BHLHE22
GAD1B
IRX7
NEUROD2
FEZF1
IRX6A
CABP5A
NFIXA
NETO1L
SOX6(2OF2)
SIX3
VSX1
CABP5B
TRPM1A
DMBX1B
ISL1
PROX1
CRX
LHX4
NEUROD4
znorm
6
4
2
0
D
Amacrine Cell Subtype Markers
RPE
Oligodendrocytes
Rods
Bipolar_Cluster_6
Bipolar_Cluster_13
Bipolar_Cluster_8
Bipolar_Cluster_7
Bipolar_Cluster_18
Bipolar_Cluster_23
Bipolar_Cluster_11
UV−Cones
Immune Cells
Bipolar_Cluster_3
Bipolar_Cluster_16
Bipolar_Cluster_24
Bipolar_Cluster_15
Bipolar_Cluster_1
Bipolar_Cluster_12
Bipolar_Cluster_14
Bipolar_Cluster_26
Bipolar_Cluster_4
Bipolar_Cluster_22
Bipolar_Cluster_20
Bipolar_Cluster_10
Differentiating Cells
Bipolar_Cluster_9
Bipolar_Cluster_2
Bipolar_Cluster_21
S−Cones
RBCs
Muller Glia
L/M−Cones
ISL1 Horizontal Cells
Bipolar_Cluster_19
Bipolar_Cluster_17
Retinal Ganglion Cells
Bipolar_Cluster_5
Bipolar_Cluster_25
LHX1A Horizontal Cells
GABAergic/PVALB5 Amacrine Cells
Glycinergic Amacrine Cells
Starburst Amacrine Cells
GABAergic Amacrine Cells
GAD1A
PVALB5
TFAP2A
CHAT
SLC18A3A
SLC32A1 (1 OF 2)
SLC6A9
GAD2
GAD1B
SLC32A1(2OF2)
PAX-6
ELAVL2
MEIS2
SLC6A1 (1 OF 2)
znorm
6
4
2
0
