# Supplementary material for: Age‐related dysregulation of the retinal transcriptome in African turquoise killifish
Source: Aging Cell. 2024 May 14;23(8):e14192. doi: 10.1111/acel.14192 (PMC11320354; doi:10.1111/acel.14192)
Supplement: Supplementary file 7 — Figure S7. [file ACEL-23-e14192-s001.zip › Figure S7.docx]

Figure S7. Heatmaps showing scRNAseq expression of cell subtype marker genes across all cell types identified. (A) Cone subtype marker genes; (B) Horizontal cell subtypes; (C) Bipolar cell subtypes; (D) Amacrine cell subtypes. Coinciding with the in situ expression pattern observed in Figure 4, barhl2 (B) transcript is detected most abundantly in horizontal cells but is also observed in ganglion and amacrine cells.
