# Supplementary material for: Age‐related dysregulation of the retinal transcriptome in African turquoise killifish
Source: Aging Cell. 2024 May 14;23(8):e14192. doi: 10.1111/acel.14192 (PMC11320354; doi:10.1111/acel.14192)
Supplement: Supplementary file 8 — Figure S8. [file ACEL-23-e14192-s011.zip › Figure S8.docx]

Figure S8. Comparison of age-related transcriptional changes between the bulk and single-cell RNAseq datasets. Boxplots indicating the age-dependent fold change of differentially expressed genes from bulk RNAseq within the scRNAseq dataset, comparing (A) Fold-change of expression comparing 12-week and 6-week-old retinas across all cell types, or (B) cell type-specific changes across those ages. (C) Boxplots indicating transcript fold change across all cell types, or (D) within individual cell types in 18-week-old retinas in comparison to 6-weeks-old fish. On average, genes displaying differential expression by bulk RNAseq analysis displayed corresponding changes within the scRNAseq dataset.
