# Supplementary material for: Age‐related dysregulation of the retinal transcriptome in African turquoise killifish
Source: Aging Cell. 2024 May 14;23(8):e14192. doi: 10.1111/acel.14192 (PMC11320354; doi:10.1111/acel.14192)
Supplement: Supplementary file 9 — Figure S9. [file ACEL-23-e14192-s009.zip › Figure S9.docx]

Figure S9. Heatmap and UMAP for genes with reduced expression between young and old killifish retinas. (A) Heatmap of integrated bulk and scRNAseq, showing the cell types that normally express those genes (left) and the changes in expression across the ages investigated for the cell types (right). (B) UMAP showing that the genes that are going down in expression with age are typically expressed in the RPE. There are several bright cells within the RPE cluster; see inset. RBCs = red blood cells, RPE = retinal pigment epithelium.
